# Pleiotropic Activity of Metformin and Its Sulfonamide Derivatives on Vascular and Platelet Haemostasis

**DOI:** 10.3390/molecules25010125

**Published:** 2019-12-28

**Authors:** Magdalena Markowicz-Piasecka, Kristiina M. Huttunen, Adrianna Sadkowska, Joanna Sikora

**Affiliations:** 1 Laboratory of Bioanalysis, Department of Pharmaceutical Chemistry, Drug Analysis and Radiopharmacy, Medical University of Lodz, ul. Muszyńskiego 1, 90-151 Lodz, Poland; joanna.sikora@umed.lodz.pl; 2 School of Pharmacy, Faculty of Health Sciences, University of Eastern Finland, Yliopistonranta 1C, POB 1627, 70211 Kuopio, Finland; kristiina.huttunen@uef.fi; 3 Students Research Group, Laboratory of Bioanalysis, Department of Pharmaceutical Chemistry, Drug Analysis and Radiopharmacy, Medical University of Lodz, ul. Muszyńskiego 1, 90-151 Lodz, Poland; adrianna.sadkowska@stud.umed.lodz.pl

**Keywords:** endothelium, vascular smooth muscle, metformin, platelet, biguanides

## Abstract

As type 2 diabetes mellitus (T2DM) predisposes patients to endothelial cell injury and dysfunction, improvement of vascular function should be an important target for therapy. The aim of this study was to evaluate the effects of metformin, its sulfenamide and sulfonamide derivatives on selected parameters of endothelial and smooth muscle cell function, and platelet activity. Metformin was not found to significantly affect the viability of human umbilical vein endothelial cells (HUVECs) or aortal smooth muscle cells (AoSMC); however, it decreased cell migration by approximately 21.8% in wound healing assays after 24 h stimulation (wound closure 32.5 µm versus 41.5 µm for control). Metformin reduced platelet aggregation manifested by 19.0% decrease in maximum of aggregation (A_max_), and 20% reduction in initial platelet aggregation velocity (*v*_0_). Furthermore, metformin decreased spontaneous platelet adhesion by 27.7% and ADP-induced adhesion to fibrinogen by 29.6% in comparison to control. Metformin sulfenamide with an *n*-butyl alkyl chain (compound **1**) appeared to exert the most unfavourable effects on AoSMC cell viability (IC_50_ = 0.902 ± 0.015 μmol/mL), while 4-nitrobenzenesulfonamide (compound **3**) and 2-nitrobenzenesulfonamide (compound **4**) derivatives of metformin did not affect AoSMC and HUVEC viability at concentrations up to 2.0 μmol/mL. These compounds were also found to significantly reduce migration of smooth muscle cells by approximately 81.0%. Furthermore, sulfonamides **3** and **4** decreased the initial velocity of platelet aggregation by 11.8% and 20.6%, respectively, and ADP-induced platelet adhesion to fibrinogen by 76.3% and 65.6%. Metformin and its *p*- and *o*-nitro-benzenesulfonamide derivatives **3**, **4** appear to exert beneficial effects on some parameters of vascular and platelet haemostasis.

## 1. Introduction

The associations between T2DM and concomitantly occurring haemostasis disturbances, including thrombosis and the activation of blood coagulation are widely known [1]. Lipiński and Pretorius [2] divide the characteristic haemostatic abnormalities in T2DM into those occurring in stages of coagulation: thrombus formation, impaired fibrinolysis, endothelial dysfunction and platelet hyperreactivity. For instance, it was reported that platelets in diabetes subjects are characterized by hyperreactivity, increased adhesiveness, exaggerated aggregation and changed metabolism [3]. T2DM patients can also present a pro-coagulation state following increased activity of coagulation factors such as fibrinogen, vWF, or factor FVII [1]. In T2DM patients, increased fibrin levels and thrombin generation have also been reported [3]. Finally, vascular endothelial dysfunction can also induce a pro-coagulant state in diabetes subjects, and can lead to the pathogenesis and clinical expression of atherosclerosis [4].

Metformin is a first-line treatment option for patients with type 2 diabetes mellitus (T2DM) due to its glucose-lowering properties [5]. The current scientific reviews indicate that in vitro laboratory experiments [1], experiments in animal [6,7] and human studies [8,9] provide supportive evidence on other beneficial effects of metformin, such as its anti-inflammatory, anti-oxidative and anti-cancer properties. The most significant breakthroughs in the history of metformin use in T2DM were summarized in the United Kingdom Prospective Diabetes Study (UKPDS) trial, which found treatment with metformin to be associated with a significant (42%) reduction of diabetes-related death and all-cause mortality (36% reduction) [10,11]. This reduction in cardiovascular mortality was also confirmed by Cochrane analysis [12]. More recently, an evaluation of 35 clinical trials including 18,472 participants treated with metformin or a comparator found that although metformin therapy did not generally induce any significant effect on the incidence of cardiovascular events compared to the active molecule, metformin monotherapy was found to improve overall mortality [13]. The findings suggest that metformin does not exert any harmful effect on cardiovascular risk, and imply a possible benefit over placebo/no treatment [13]. There is also a growing body of evidence that metformin attenuates ischemia-reperfusion injury in myocardium and prevents remodelling induced by humoral and hemodynamic factors [14,15]. These effects may be attributed to the beneficial influence of metformin on endothelial function, oxidative stress, inflammation and the negative effects of angiotensin II, and that it inhibits the proliferation of smooth muscle cells [15].

Therapy with metformin has also been found to offer a moderate anti-hypertensive [16,17], anti-atherosclerotic [18] and pro-fibrinolytic benefits [19]. The main mechanisms for the atheroprotective effect of metformin are believed to involve the inhibition of leukocyte-endothelial interaction, foam cell formation, smooth muscle cell proliferation and platelet aggregation [20,21].

Metformin positively affects the viability and functions of the endothelium [22]; for instance, it has also been found to markedly improve endothelial-dependent vasodilation, and simultaneously reduce the expression of dysfunctional biomarkers such as endothelin-1 (ET-1), plasminogen activator inhibitor-1 (PAI-1), and C-Reactive protein (CRP) in endothelial cells [23]. A great number of preclinical and clinical studies also confirm that metformin has positive effects on endothelial function [24,25]. It has also been shown that treatment with metformin is associated with improvement in some markers of endothelial functions, such as von Willebrand factor (vWF) and vascular cell adhesion molecule 1 (VCAM-1) [26]. Additionally, metformin protects from oxidative stress and inflammation, and the negative effects of angiotensin II [27].

Some reports describe the effects of metformin on vascular smooth muscle cells, whose proliferation is a well-known etiological factor of atherosclerosis, restenosis, and pulmonary hypertension [28]. For instance, it was found that metformin appears to attenuate cardiac remodelling by reducing smooth muscle cell proliferation, hypertrophy, and inflammation-induced damage [15]. Mounting evidence indicates that metformin antagonizes the proliferation and migration of human aortal smooth muscle cells (HASMCs) through activation of AMPK. For example, Hao et al. [29] report that treatment with metformin can significantly inhibit the growth and migration of HASMCs. However, it should be noticed that these beneficial activities were reported for very high metformin concentrations (reaching 10 mmol/L) which are impossible to achieve in vivo.

Although a number of reports confirm that metformin exerts beneficial effects on the cardiovascular system, only a few studies examine the impact of metformin on platelets. Furthermore, most of these studies have generally been reported as a component of other studies and do not tackle the issue systematically [16]. For instance, a study by Formoso et al. [30] among 26 newly diagnosed T2DM patients found that metformin improved oxidative stress, preserved antioxidant function and decreased platelet activation.

Despite displaying promising and multidirectional pharmacological properties, metformin is characterized by an unfavourable pharmacokinetic profile: slow and incomplete absorption from the gastrointestinal tract [31]. Therefore, the synthesis of novel biguanide-based compounds to enhance bioavailability appears to be justified. Sulfonamide-based compounds (Figure S1, Supplementary Materials) were found to exhibit beneficial effects on plasma, vascular and platelet haemostasis, which is frequently impaired in T2DM patients [32,33,34]. For instance, *o*-nitrobenzenesulfonamide was found to demonstrate more prominent effects on certain plasma and vascular haemostasis parameters than the parent drug metformin [32,33,34]. The beneficial effects of sulfenamide and sulfonamide derivatives of metformin on plasma and vascular haemostasis have been summarized in Figure S1 (Supplementary Materials).

**Figure 1:**
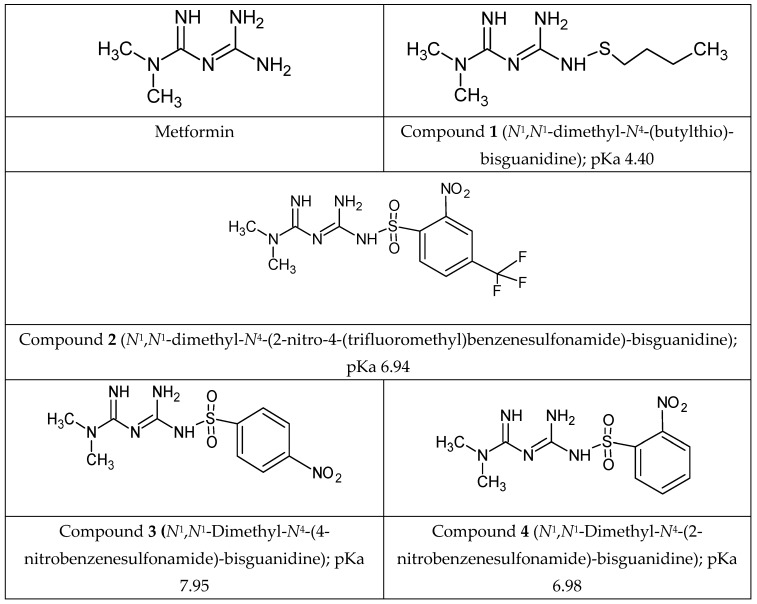
Chemical structure of tested biguanide derivatives – metformin, and compounds **1**–**4**. Compound **1**-*N*^1^,*N*^1^-dimethyl-*N*^4^-(butylthio)-bisguanidine; Compound **2**-*N*^1^,*N*^1^-dimethyl-*N*^4^-(2-nitro-4-trifluoromethylbenzenesulfonamide)-biguanidine, Compound **3**-*N*^1^,*N*^1^-dimethyl-*N*^4^-(4-nitro-benzenesulfonamide)-biguanidine, Compound **4**-*N*^1^,*N*^1^-dimethyl-*N*^4^-(2-nitrobenzenesulfonamide)-biguanidine.

The anti-coagulation properties of some derivatives, especially nitrobenzene sulfonamides, encouraged us to undertake further studies, including the effects on human aortal smooth muscle cells, and platelet haemostasis. We have selected the most promising derivatives, including *n*-butyl sulfenamide, and nitrobenzenesulfonamides (Figure 1) to broaden the knowledge on their effects on the viability, function and migration of aortal smooth muscle cells, and to compare their properties with parent drug, metformin. Since intercellular adhesion molecule 1 (ICAM-1) participates in migration of endothelial and vascular smooth muscle cells [35,36] we decided to determine the effects of metformin derivatives on ICAM-1 expression on SMC.

The scientific value of the current manuscript relates also to the effects of metformin, and its derivatives on the kinetic parameters of ADP-induced platelet aggregation, and platelet adhesion to fibrinogen. The key findings of the current study together with our previous results [34,37] indicate that chemical modification of biguanide backbone into sulfonamides with nitrobenzene substituent contributes to the formation of potential agents with stronger anti-coagulant properties than the parent drug, metformin, and can be regarded as an initial promising step in the development of novel biguanide-based compounds with anti-coagulant properties. Considering the varied multi-directional effects of metformin on the cardiovascular system and coagulation, and beneficial effects of metformin derivatives on plasma haemostasis, this paper presents the effects of metformin, and its selected sulfenamide and sulfonamide derivatives (Figure 1) on the viability of human umbilical vein endothelial cells (HUVECs), and human aortal smooth muscle cells (AoSMCs) using cellular in vitro models. To further characterise the mode of action of biguanides in AoSMC cell apoptosis, and intercellular adhesion molecule 1 (ICAM-1) expression on their surface were determined. The influence of biguanide derivatives on the inhibition of smooth muscle cells migration was also established. Finally, the paper evaluates the influence of metformin derivatives on the ADP-induced aggregation and adhesion of platelets.

## 2. Results

### 2.1. Cell Viability Studies

**Figure 2:**
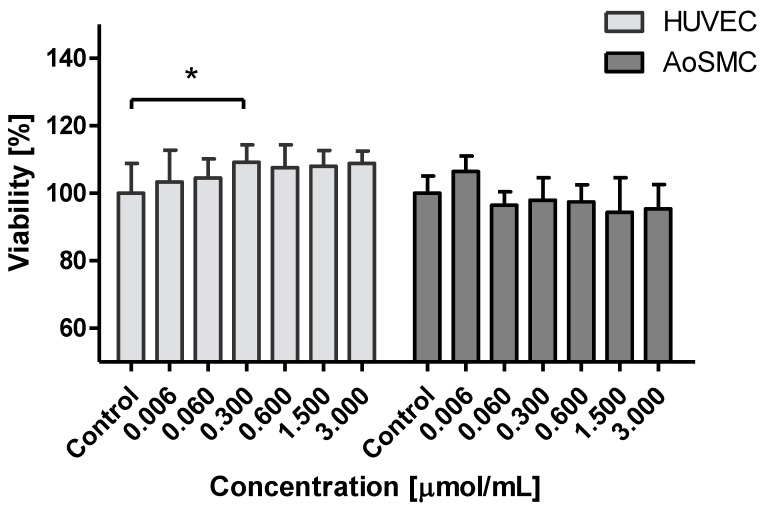
The effects of metformin on the viability of HUVEC and AoSMC cells. The cells were incubated with metformin at concentrations 0.006–3.000 μmol/mL for 24 h, and the viability of the cells was measured spectrophotometrically using the WST-1 test. The results are presented as mean ± SD, *n* = 8. One-way Anova analysis revealed significant differences in HUVEC viability only for metformin at 0.300 µmol/mL (* *p* < 0.05). In the case of AoSMC cells the analysis did not show any differences between control samples and metformin over the entire concentration range.

The effects of the biguanide derivatives on the viability of two human cell lines, HUVEC and AoSMC, were determined using WST-1 assay, which measures mitochondrial function. Metformin was found to increase the viability of HUVEC cells, but only 0.3 µmol/mL gave a significant change. In the case of AoSMC cells, metformin did not induce any significant effects on cell viability up to a concentration of 3.0 µmol/mL (Figure 2).

The other compounds showed concentration-dependent effect on HUVECs and AoSMCs viability, and for most of other tested compounds, a concentration-response analysis was performed to determine the concentration inducing a 50% decrease of cell viability (IC_50_) (Table 1). The effects of compounds **1**–**4** at various concentrations ranging from 0.006 µmol/mL to 3.0, 5.0 or 10.0 µmol/mL, depending on the compound, and cell line, on the viability of both cell lines are presented in Figures S2 and S3 (Supplementary Materials).

As presented in Table 1, compound **1**, with an *n*-butyl chain, inhibited AoSMC cell viability with IC_50_ values of 0.902 ± 0.015 µmol/mL. In contrast, compound **1** was far less toxic towards HUVECs since its IC_50_ value in these cells was 3.781 ± 1.130 µmol/mL. Sulfonamide **2** with the CF_3_ substituent was found to demonstrate comparable growth inhibition on both cell lines (IC_50_ = 1.044 ± 0.131 µmol/mL for HUVECs, and IC_50_ = 1.247 ± 0.062 µmol/mL for AoSMCs). Both *o*- and *p*- nitrosulfonamides appear not to affect HUVEC and AoSMC cell growth up to a concentration of ca. 1.5–2.0 µmol/mL, however, they appear to be less toxic towards AoSMC cells since at the concentration of 3.0 µmol/mL they decreased viability up to 79.33%, and 62.19%, respectively. The results of HUVECs and AoSMCs viability determined the concentrations of studied compounds in further experiments, including apoptosis assay, migration test and ICAM-1 expression. On the basis of the conducted viability assay we can indicate that compounds **1** and **2** are much more toxic to HUVEC and AoSMC cells, therefore we decided to test them up to the concentration of 1.0 µmol/mL. Nitro-benzenesulfonamides (compounds **3** and **4**) are also characterized by higher toxicity towards both cell lines than the parent drug, metformin. However, these compounds up to 1.5 µmol/mL appear to exert weak or moderate effects on cells viability (Table 1, Figure S2, Figure S3). These results are of vital importance due to our previous results suggesting highly demanded anti-coagulation properties of these compounds at the concentrations of 0.3–1.5 µmol/mL [34]. A previously published study [34] using the RTCA-DP system has shown that sulfonamides **3** and **4** are able to compensate for the immediate cell monolayer reaction and during 36-h incubation do not lead to a significant reduction in the integrity and barrier properties of endothelial cells and smooth muscle cells.

### 2.2. Apoptosis

**Figure 3:**
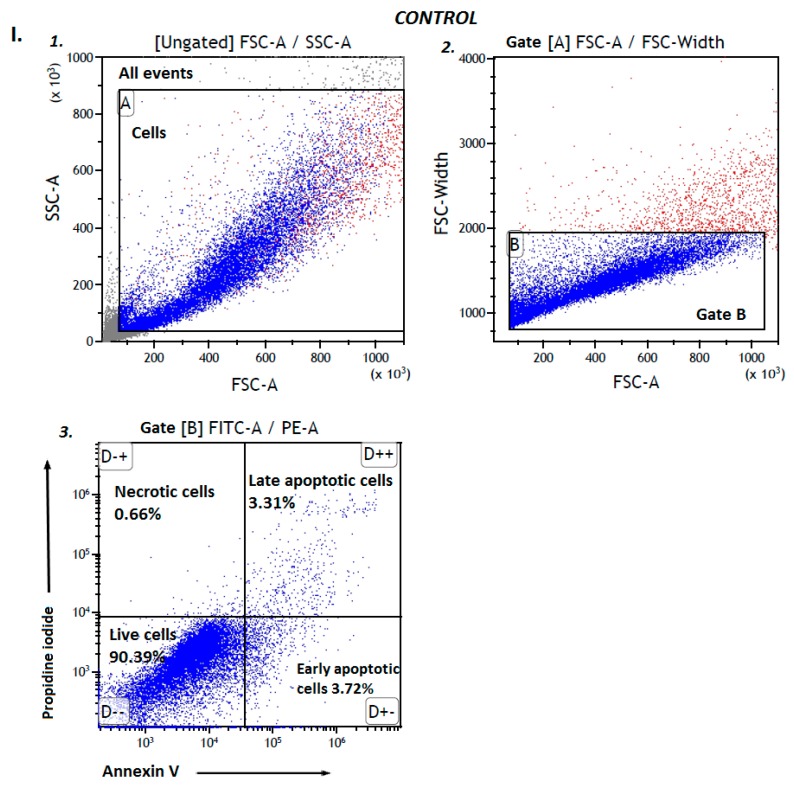

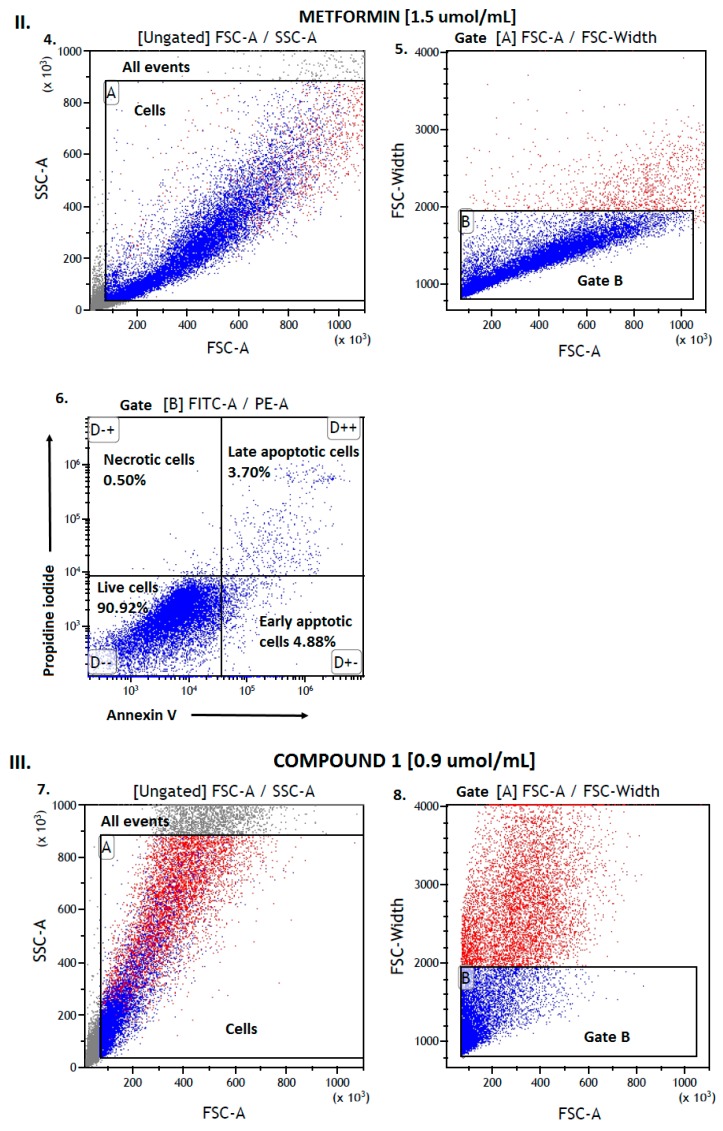

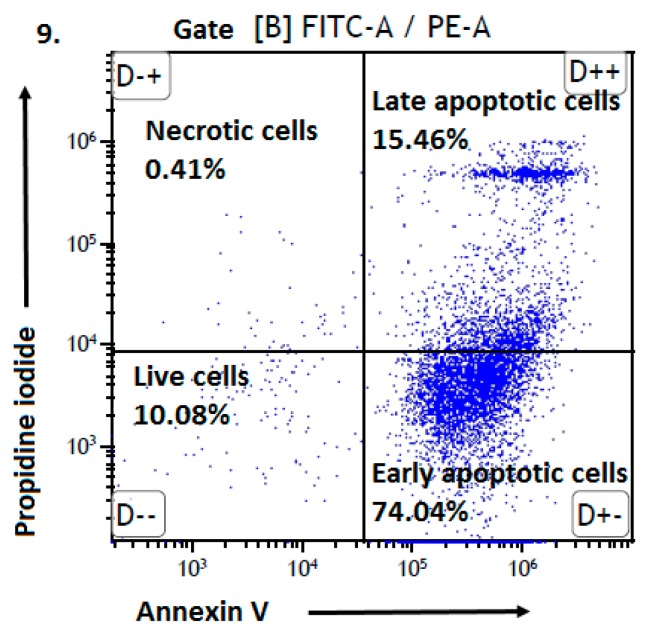
The effect of selected biguanides on AoSMC viability. (**I**) Representative (exemplary) cytograms of unstimulated AoSMCs (control). Cytogram 1. - Forward and side scatter plot of AoSMC control sample. The cells were marked with gate A. Cytogram 2.-A forward scatter width (FSC-W) vs. forward scatter area (FSC-A) of the cells gathered in gate A. Based on FSC-A parameters, single cells were divided, and marked with gate B. The single cells in gate B were analysed for staining with Annexin V and propidine iodide. Cytogram 3. - Annexin V (x-axis) vs propidium iodide (y-axis) plots from the gated cells (B) show the populations corresponding to living cells (Annexin V(-) and PI (-)) (gate D- - on cytogram); early apoptotic cells (Annexin V (+) and PI (-)) (gate D+- on cytogram), late-apoptotic cells (Annexin V(+) and PI (+)) (gate D++ on cytogram), and necrotic cells (Annexin V (-) and PI (+)) (gate D-+ on cytogram). (**II**) Representative (exemplary) cytograms of AoSMCs stimulated with metformin at the concentration of 1.5 μmol/mL. Cytogram 4. - Forward and side scatter plot of AoSMC cells stimulated with metformin. The cells were marked with gate A. Cytogram 5.-A forward scatter width (FSC-W) vs. forward scatter area (FSC-A) of the cells gathered in gate A. The single cells in gate B were analysed for staining with Annexin V and propidine iodide. Cytogram 6. - Annexin V (x-axis) vs propidium iodide (y-axis) plots from the gated cells (B) show the populations corresponding to living cells (gate D—on cytogram); early apoptotic cells (gate D+- on cytogram), late-apoptotic cells (gate D++ on cytogram) and necrotic cells (gate D-+ on cytogram). (**III**) Representative cytograms of AoSMCs treated with compound **1** at a concentration of 0.9 μmol/mL. Cytogram 7. - Forward and side scatter plot of AoSMC cells treated with compound **1**. The cells were marked with gate A. Cytogram 8. - A forward scatter width (FSC-W) vs. forward scatter area (FSC-A) of the cells gathered in gate A. The single cells in gate B were analysed for staining with Annexin V and propidine iodide. Cytogram 9.-Annexin V FITC-A (x-axis) vs PI (y-axis) plots from the gated cells (B) show the populations corresponding to living cells (gate D—on cytogram), early apoptotic cells (gate D+- on cytogram), late-apoptotic cells (gate D++ on cytogram) and necrotic cells (gate D-+ on cytogram).

As vascular smooth muscle cells demonstrate abnormal growth and apoptosis in vascular dysfunction, damage and the premature development of atherosclerosis [36], an apoptosis assay was performed to further characterize the mode of action of biguanide derivatives on AoSMC cells. AoSMC cells were incubated with the tested compounds and then stained with propidine iodide (PI) and Annexin V (AV) for flow cytometry (Figure 3, Table 2). Metformin was found to not affect the number of the cells gathered within gates B (single events, according to FSC-a parameter) at either concentration (0.3 and 1.5 µmol/mL) (Table 2). Metformin concentrations were chosen on the basis of data presented by Kajbaf et al. [38], who found that therapeutic plasma concentration of metformin is 0.8 nmol/mL–0.6 µmol/mL. We decided to exceed 2.5-fold this concentration since in the literature there are many examples of in vitro studies testing metformin even at 10–50 mM range [39]. Similarly, stimulation of AoSMCs with metformin did not change the percentage of viable cells (Annexin V–(AV) propidine iodide–(PI-) constant). The other compounds were tested at concentrations relating to their effects on cells viability. For instance, compound **1** was examined using the concentration reflecting its IC_50_ value towards AoSMC cells (IC_50_ = 0.902 ± 0.015 µmol/mL). Unlike metformin, compound **1** at a concentration of 0.90 µmol/mL contributed to a significant decrease in the percentage of cells within gate B (gate gathering only the single cells events). Following incubation with compound **1,** the percentage of living cells decreased to 12.33 ± 2.03%. The analysis of the cells in gate B showed a significant increase in early- and late-apoptotic cells (increased number and percentage (↑) of AV + PI-; increased (↑) AV + PI+). In the case of 1.0 µmol/mL compound **2,** a significant decrease in the number of viable cells (decreased (↓) number of AV–PI-) was also observed.

Compounds **3** and **4** were tested at two concentrations 0.3 and 1.5 μmol/mL due to the moderate effects on AoSMC cells viability, and for convenient comparison with metformin effects. Compound **3** at both tested concentrations was not found to exert significant effects on the percentage of early- and late- apoptotic cells. However, the percentage of viable cells treated with compound **4** was reduced in comparison with control (↓AV–PI-) at both tested concentrations; in the case of 1.5 µmol/mL, the population of early- and late-apoptotic cells was increased (↑AV+PI-; ↑AV+PI+). On the other hand, compound **4** does not contribute to the necrosis of AoSMC cells. Most of the current literature concentrates on the effects of metformin on the apoptosis of endothelial cells [40,41]. Therefore, this is one of the first studies reporting the effects of metformin and its sulfonamide derivatives on viability and apoptosis of vascular smooth muscle cells.

### 2.3. Migration Test

Vascular smooth muscle cells, constituting the medial layer of the artery wall, play a crucial role in the physiological functions of the blood vessels, such as vasoconstriction and vasodilatation, but also in the pathogenesis of vascular diseases, particularly hypertension and atherosclerosis, in which increased apoptosis and expression of intercellular adhesion molecule-1 (ICAM-1) are observed [36]. During atherogenesis, smooth muscle cells migrate to populate the intima, which finally lead to vascular wall remodelling. Inhibition of vascular smooth muscle cell migration and proliferation might be useful for preventing or reducing atherogenesis. Vessel wall remodelling can be antagonized by some cardiovascular drugs, including statins [42]. There is also some evidence from experimental in vitro and in vivo studies, showing that metformin exerts beneficial effects on vascular function, and these are partly independent of its hypoglycaemic effects. Therefore, the present study examines the effects of metformin and its derivatives on aortal smooth muscle cell migration.

**Figure 4:**
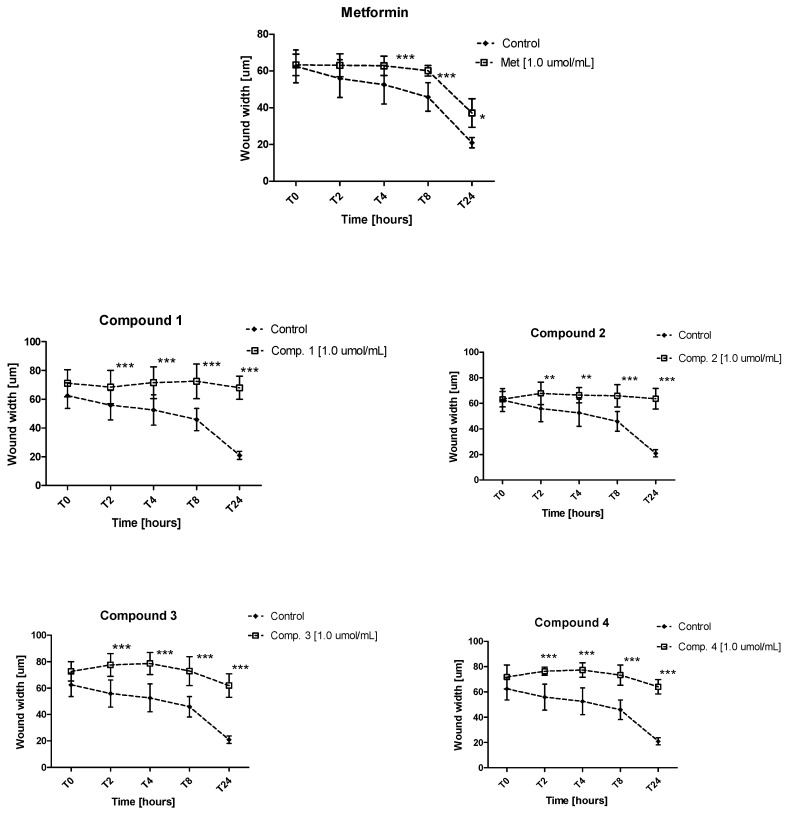
Inhibition of cell migration in the presence of biguanides. AoSMC cell migration was evaluated using wound healing assay. Graphs depict the changes of wound width [µm] during 24 h in the absence (control) and in the presence of examined compounds at 1.0 μmol/mL. The results are presented as mean ± SD (*n* = 6–10). *p < 0.05; ** *p* < 0.01; *** *p* < 0.001 compared with control. Two-way Anova analysis exhibited more profound effects of compounds **1** and **2** on AoSMC cell migration than compounds **3** and **4**.

The potential of metformin and its derivatives to reduce cell migration was investigated using in vitro wound healing assay. The cells were seeded on 24-well plates for 24 h; a wound was made, and then co-treated with various concentrations of tested compounds. The ability of compounds to affect AoSMC cell migration was monitored microscopically after 2, 4, 8 and 24 h of stimulation. The potential for biguanides to attenuate cell migration is presented in Table S1 (Supplementary Materials). Figure S4 (Supplementary Materials) shows representative images of wound closure at the starting point, 8 and 24 h of stimulation with metformin, and other compounds. Metformin was found to significantly modulate the cell migration, indicated by an increase in the width of the wound in comparison with control over the whole concentration range (0.06–1.5 μmol/mL) (Figure 4, Table S1). Similar results were obtained by Esfahanian et al. [22], who reported that cell proliferation and migration were markedly inhibited by metformin. Metformin was also found to demonstrate inhibitory properties towards proliferation and migration in cardiac fibroblasts.

Migration tests showed that all derivatives **1**–**4** significantly inhibited smooth muscle cell migration. The greatest effect on wound width was observed after 24 h of incubation (Table S1, Figure 4). The highest concentration of compounds **1** and **2** was 1.0 μmol/mL, which contributed to significant inhibition of AoSMCs migration at all time points (2, 4, 8 and 24 h), however, these effects could be attributed to the decreased viability of the cells. Lower concentrations of these both compounds (0.06–0.3 μmol/mL) have also contributed to the reduced smooth muscle cells migration, but these effects were not statistically significant at all time points. As presented in Table S1 both nitro-benzenesulfonamides significantly prevented AoSMC cell migration over the whole concentration range. The results obtained in migration assay provide information on the valuable properties of examined biguanides, since pathological migration is a major factor in atherogenesis and restenosis [43].

### 2.4. Effects on ICAM-1 Expression

**Figure 5:**
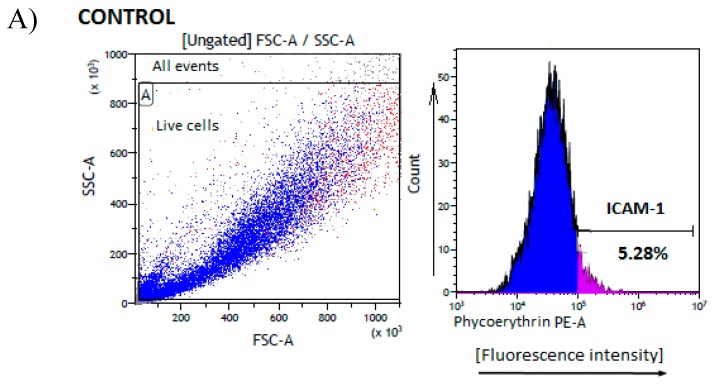

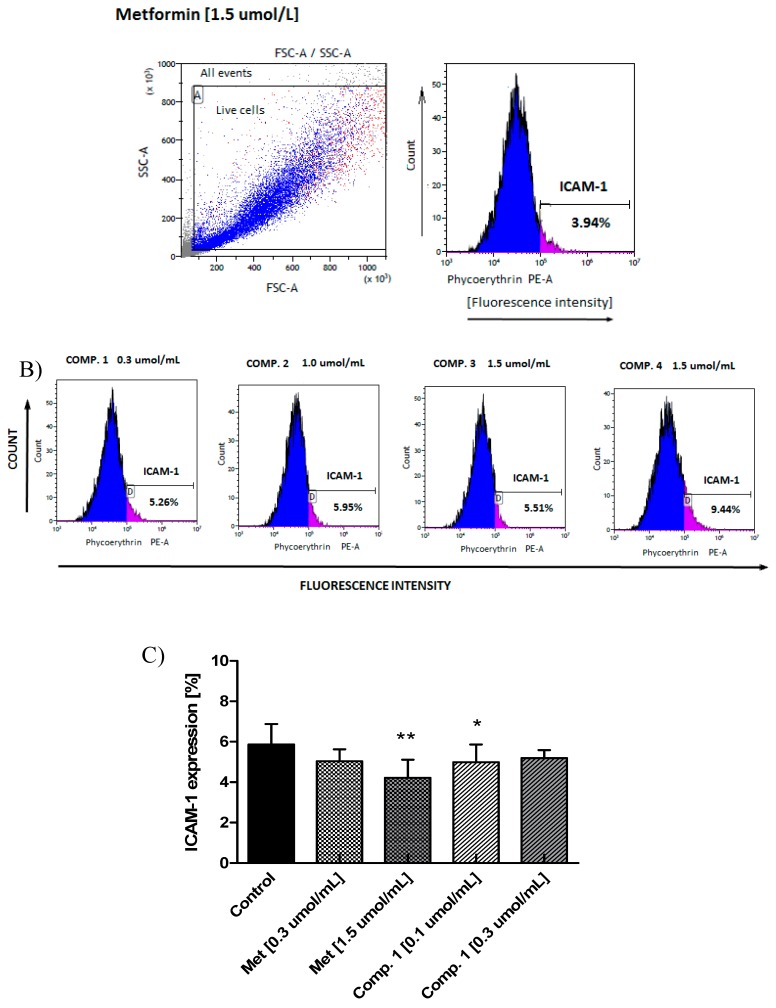

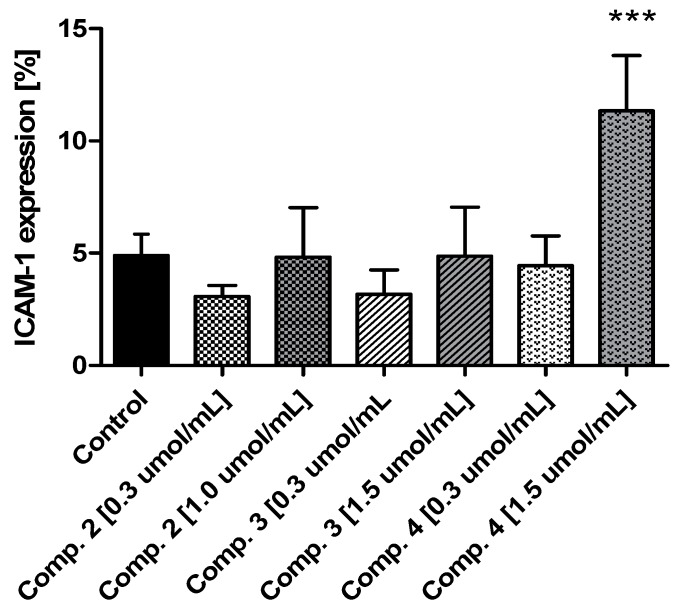
The effect of metformin and its derivatives on ICAM-1 expression on AoSMCs. (**A**) Flow cytometry analysis of the expression of ICAM-1 on the surface of AoSMC cells. Representative histograms of unstimulated AoSMC cells (control–CD54 [CTR] and isotype control [CTR-isot]) and cells treated with metformin (1.5 μmol/mL). Cytograms on the left side: forward and side scatter plot of AoSMC cells. The living cells were marked with gate A. Cytograms on the right side: percentage of single cells expressing ICAM-1. (**B**) The effects of compounds **1**–**4** administered at the highest concentrations on the percentage of the surface ICAM-1 expression (representative histograms). (**C**) The summary of effects of metformin, and compounds **1**–**4** on ICAM-1 expression (*n* = 3–6), mean ± SD;* *p* < 0.05; ** *p* < 0.01; *** *p* < 0.001. Two-way Anova analysis revealed significantly greater ICAM-1 expression on AoSMC cells surface in the case of compound **4** at the concentration 1.5 µmol/mL.

Within this paper we also evaluated the influence of biguanides on the expression of the ICAM-1 adhesion molecule on AoSMC cells. These intercellular molecules serve as adhesion particle for leucocytes and facilitate SMC migration [36]. Of vital importance, adhesion molecules are cell cycle dependent and influence smooth muscle cells proliferation and differentiation [44]. Furthermore, ICAM-1 are engaged in the development of atherosclerosis, and play an important role in stabilizing cell-cell interactions [36]. The data regarding the effects of biguanides on the expression of ICAM-1 on AoSMCs is given in Table S2, Supplementary materials. Both tested concentrations of metformin decreased the expression of ICAM-1 on the surface of AoSMCs in comparison to unstimulated cells; however, statistically significant changes were only reported for 1.5 µmol/mL (Figure 5A,C). Some studies have presented the effects of metformin on ICAM-1 expression on various types of cells. For instance, Zhang et al. [45] showed that metformin down-regulates ICAM-1, blocks neutrophil infiltration and attenuates blood-spinal cord barrier (BSCB) permeability after spinal cord injury (SCI) in rats. However, our previous experiments revealed that metformin does not affect ICAM-1 expression on HUVECs [34].

Compound **1** was tested at lower concentrations in comparison to other tested biguanides to avoid the unfavourable effects on cell viability, and consequently falsely lower ICAM-1 expression. Compound **1** at 0.1 and 0.3 µmol/mL was not shown to influence ICAM-1 expression (Figure 5B,C). Similarly, compounds **2** and **3** did not affect the expression of ICAM-1 at either tested concentration. Only 1.5 µmol/mL compound **4** significantly increased the expression of ICAM-1 on AoSMC surface (Figure 5B,C). Since the induction of adhesion molecules is much weaker in proliferating cells compared to quiescent cells [44] we presume that increased ICAM-1 expression in AoSMC cells treated with compound **4** at 1.5 µmol/mL might stem from its effects on cellular viability and apoptosis induction.

### 2.5. Platelet Aggregation

**Figure 6:**
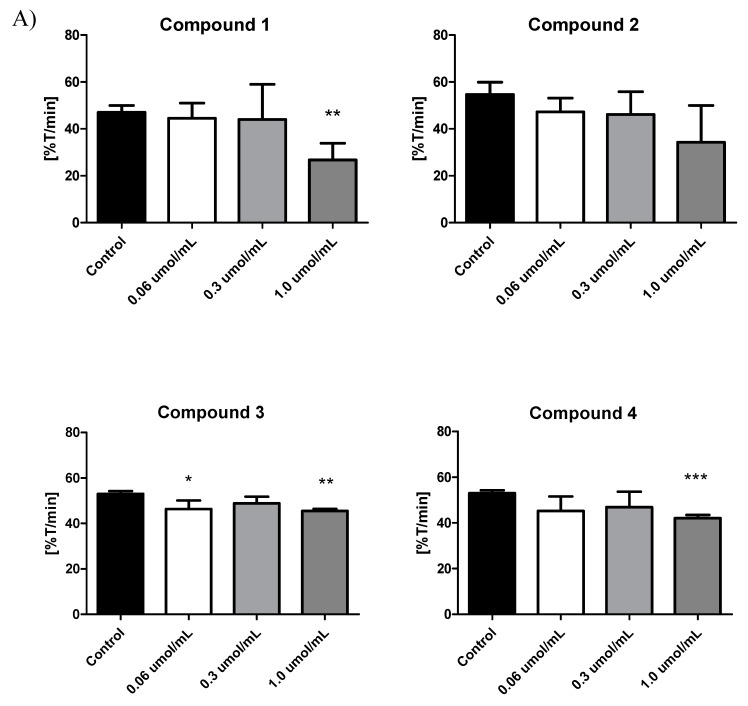

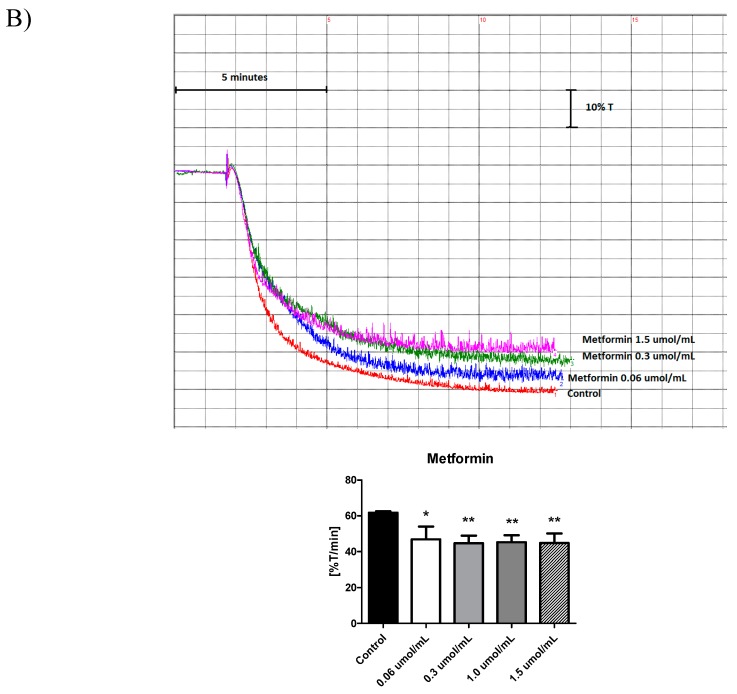
In vitro effect of biguanide derivatives on ADP-induced platelet aggregation. (**A**) The effects of compounds **1** – **4** on the initial platelet aggregation velocity (*V*_0_). The results are presented as mean ± SD, *n* = 4–5. * *p*< 0.05; ** *p* < 0.01; *** *p* < 0.001. In the case of compound **3** at the concentration of 0.3 µmol/mL, the *p*-value was 0.059. Two-way Anova analysis found 1.0 µmol/mL compound **1** to possess the most significant inhibitory effect on platelet aggregation. (**B**) The effect of metformin on the representative curve of platelet aggregation (left): 1-control, 2-metformin at 0.06 µmol/mL, 3-metformin at 0.3 µmol/mL, 4-metformin at 1.5 µmol/mL. The bold line represents 5 min and 10% of transmittance (T). The effect of metformin on the initial platelet aggregation velocity (*V*_0_) (right). The results are presented as mean ± SD, *n* = 4–5. * *p* < 0.05; ** *p* < 0.01.

It has been established that platelets play a pathogenic role in the formation and maintenance of thrombi. Additionally, in the case of T2DM, hyperglycaemia and insulin resistance increase platelet reactivity [46]. Although a number of reports confirm that metformin exerts beneficial effects on the cardiovascular system, only a few studies examine the impact of metformin on platelets. Furthermore, most of these studies have generally been reported as a component of other studies and do not tackle the issue systematically [16]. Therefore, we have conducted the studies on the effects of biguanide derivatives on ADP-induced platelet aggregation. The effects of metformin and its four derivatives on the kinetic parameters of ADP-induced platelet aggregation is presented in Table S3 (Supplementary Materials). Incubation of PRP with metformin significantly decreased initial velocity (*v*_0_) of aggregation process (Table S3, Figure 6B). Behind these, maximum aggregation (A_max_) was also decreased; however, these changes were not of statistical significance. These results suggest that the anti-thrombotic effects of metformin obtained in T-TAS experiments [34] may be attributed to its anti-aggregatory properties, expressed as decreased initial velocity of aggregation (*p* < 0.01) and reduced maximum aggregation (A_max_, NS). These results are in agreement with those of Gin et al. [47] who also found that metformin decreases maximum platelet aggregation induced by ADP in vitro. Also Formoso et al. [30] found that metformin decreased platelet activation in newly diagnosed T2DM patients.

The *n*-butylsulfenamide derivative of metformin (compound **1**) exerted an inhibitory effect on kinetic parameters of the aggregation process, expressed by decreased v_0_, A_max_ and A_5min_ (Table S3, Figure 6A). In the case of sulfonamide derivatives, a significant decrease in v_0_ was observed (Figure 6A) apart from compound **2**. For instance, compound **4** (1.0 µmol/mL) reduced A_max_ by ca. 23%, and v_0_ of the process by approximately 25%. These effects might account for the observed prolongation of platelet thrombus formation (↑ OT, ↑ CT) and decreased area under curve (AUC) values for clot formation in T-TAS experiments. In addition to its anti-aggregatory potential, compound **4** was found to significantly decrease the overall potential of clot formation and fibrinolysis (↓ CL_AUC_), prolong PT and APTT and prolong fibrin-rich platelet thrombus formation under blood flow conditions [34].

### 2.6. Platelet Adhesion

Platelet adhesion in a broad meaning is the study of the adhesive properties of platelets. The process of platelet adhesion is an essential factor in response to vascular injury and includes the binding of single platelets to cellular and extracellular matrix constituents of the vessel wall and tissues [48]. Since T2DM is associated with impairments in platelets function, therefore it is important whether novel compounds do not affect this process unfavourably. Platelet adhesion was conducted on fibrinogen coated microplates, and the number of adherent platelets was determined by measuring the activity of acid phosphatase, an enzyme whose activity remains stable independent of platelet stimulation.

**Figure 7:**
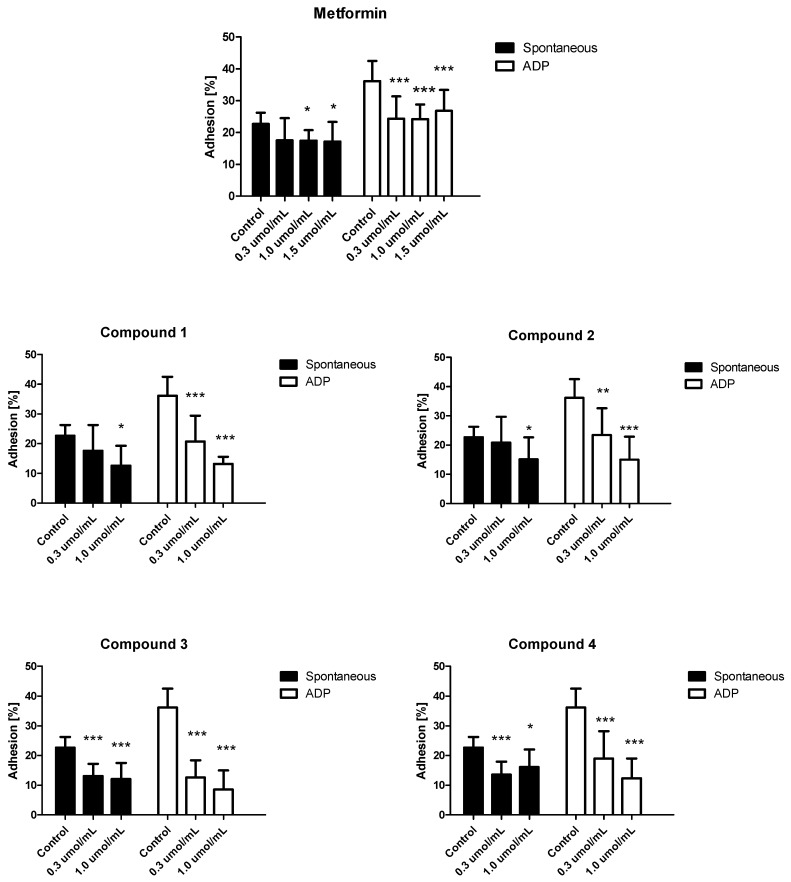
In vitro effect of metformin and its derivatives (**1**–**4**) on spontaneous and ADP-induced platelet adhesion (%). The results are presented as mean ± SD, *n* = 9–12. * *p* < 0.05; ** *p* < 0.01; *** *p* < 0.001. Two-way Anova analysis did not reveal any significant differences between the tested compounds with regard to their effects on spontaneous or ADP-induced platelet adhesion.

Incubation of PRP with metformin at a concentration of 1.0 and 1.5 µmol/mL significantly reduced spontaneous adhesion to the fibrinogen-coated surface (Figure 7). In the case of ADP-induced adhesion, significant differences were observed for the entire concentration range. Similar effect was also observed for tested biguanide derivatives. Significant decreases in spontaneous platelet adhesion were observed at higher concentrations for compounds **1** and **2**, and at 0.3 µmol/mL for compounds **3** and **4**. Moreover, all tested compounds reduced ADP-induced platelet adhesion. For instance, compound **1** with *n*-butyl chain at 0.3 µmol/mL decreased adhesion by approximately 43% (36.14 ± 6.38% for control vs. 20.73 ± 8.69). At the higher concentration (1.0 µmol/mL), compound **1** contributed to a 63% reduction in ADP-activated platelet adhesion (36.14 ± 6.38% vs. 13.15 ± 2.41% of platelets introduced into the well). The results of these studies demonstrate that inhibition of platelet interactions with fibrinogen holds great promise as an additional favourable activity of metformin and novel biguanides providing highly significant anti-coagulant properties.

## 3. Materials and Methods

### 3.1. Materials

Human umbilical vein endothelial cells (HUVEC) were purchased from Lonza (Basel, Switzerland) (3rd passage), human aortal smooth muscle cells (AoSMC) were purchased from ScienCell Research Laboratories (Carlsbad, CA, USA) (3rd passage). HUVECs were cultured using Endothelial Cell Growth Medium (EGM-2) - medium + bullet kit (Lonza, Clonetics, Basel, Switzerland), trypsin-EDTA – 0.05% solution (Sigma Aldrich, Munich, Germany), trypsin neutralizing solution (Lonza), and HEPES buffered saline solution (Lonza). SMC medium consisting of 500 mL of basal medium, 10 mL of fetal bovine serum (FBS, ScienCell Research Laboratories, US), 5 mL of smooth muscle cell growth supplement (SMCGS, ScienCell Research Laboratories), and 5 mL of penicillin/streptomycin solution (ScienCell Research Laboratories) were used for AoSMCs culturing. For passaging AoSMC cells trypsin/EDTA (ScienCell Research Laboratories), Trypsin Neutralizing Solution (ScienCell Research Laboratories), and poly-L-lysine (ScienCell Research Laboratories) were used. Cells viability was assessed using WST-1 assay (Takara, Takara Bio Europe, Saint-Germain-en-Laye, France).

Apoptosis of AoSMCs was examined using FITC Annexin V Apoptosis Detection Kit with propidine iodide (PI) (BioLegend, Kentish Town, UK) and cell staining buffer (BioLegend). The kit consists of FITC Annexin V reagent (0.5 mL), propidine iodide solution (1 mL), and Annexin V Binding Buffer (50 mL). The procedure for staining the cells was as follows, washing cells twice with cold BioLegend’s Cell Staining Buffer, and then resuspension of cells in Annexin V Binding Buffer in a volume of 100 µL in a 1.5 mL test tube, addition of 5 µL of FITC Annexin V, and 10 µL of propidium iodide solution, gentle vortexing the cells and incubation for 15 min at room temperature (25 °C) in the dark. The final step included addition of 400 µL of Annexin V Binding Buffer to each tube, and then immediate analysis by flow cytometry.

For the measurements of ICAM-1 surface expression on AoSMCs, PE anti-human CD54 Antibody (BioLegend) and PE Mouse IgG1, kappa Isotype Ctrl FC (BioLegend) were used.

Adenosine 5′-diphosphate (ADP, Sigma Aldrich, Germany), human fibrinogen (Sigma Aldrich), 0.9% NaCl (Polish Chemical Reagents, Gliwice, Poland), p-nitrophenyl Phosphate (Sigma Aldrich), and NaOH (Polish Chemical Reagents) were used in platelets activity studies.

### 3.2. HUVEC and AoSMC Cells Subculturing

HUVEC and AoSMC cells were subcultured according to the manufacturer’s guidelines (Lonza and ScienCell Research Laboratories, respectively). HUVECs between passages 3–4, and AoSMCs between 2–3 passages were used in the experiments. The morphology of both cell lines was monitored during the culturing of the cells and the experiments. Flow cytometry preliminary studies showed one homogenous population of the cells (both HUVECs and AoSMCs), not differing in SSC and FSC parameters, and viability reaching approximately 90%. The characteristic of HUVEC cells is presented on Figure S5 (Supplementary Materials).

Both cell lines were cultured in standard conditions (37 °C, 5% CO_2_, 90% humidity in incubator (HeraCell iVios160, ThermoFisher Scientific, Waltham, MA, USA). The cells were seeded in 75 cm^2^ culture T-flasks using 15 mL of medium (EGM-2 for HUVECs, SMC for AoSMCs). The cells were seeded at the density of 5000 cells per 1 cm^2^. Both cell lines (HUVECs and AoSMCs) are guaranteed to further expand for 15 population doublings under the conditions specified by Lonza and ScienCell Research Laboratories, respectively. The average doubling time for HUVECs was estimated as 26–30 h, while in the case of AoSMC it was 32–36 h. Cryopreserved HUVECs and AoSMCs vials (>500,000 cells) were thawed in water bath (37 °C) for 2 min, and the cells were transferred to culture vessel for multiplication, which took 5–6 doubling times. Then the cells were detached, counted and some of them were frozen, and stored in liquid nitrogen. Before the experiments, the cells were thawed and cultured to obtain the sufficient number of cells (2–3 doubling times). Once the cells in culture vessels reached 70% confluence they were trypsinized, neutralized with TNS solution, counted and seeded at the appropriate density on multi-well plates.

### 3.3. Tested Compounds

The subject of this study was metformin, its *n*-butylsulfenamide derivative and three sulfonamide derivatives (Figure 1). The design and syntheses of metformin derivatives **1**–**4** were performed at the University of Eastern Finland according to the protocols published earlier [49,50,51]. Brief synthesis protocol and spectroscopic characterization is included in Supplementary materials. The compounds were purified using flash chromatography.

Metformin, and its derivatives **1**–**4** were tested at the concentration range 0.006–1.5 μmol/mL, based on therapeutic plasma concentration of metformin (0.129 to 90 mg/L [38], which is 0.8 nmol/mL–0.6 µmol/mL) as well as our previous studies [32,34]. The exception was cell viability studies, where higher concentrations were used to determine IC_50_ values. The compounds were tested at this broad concentration range since we aimed to discover whether their activity could also occur at lower, pharmacologic concentrations or only at higher one, obtained only in vitro. During controlled clinical studies of metformin, plasma metformin levels generally do not exceed 5 mg/mL, which is 30 nmol/mL. Therefore we decided to start testing the compounds at 5-fold lower concentration (6 nmol/mL). In turn, high metformin concentrations (doses) are also widely employed in animal and in vitro studies [39], thus we decided to extend the concentrations up to 1.5 μmol/mL. Some of the authors examine metformin biological activity even at much higher concentrations, reaching 10 or 50 mmol/L, however, we did not want to test the compounds at concentrations exceeding 100-fold those clinically relevant.

### 3.4. Cell Viability Studies

HUVECs and AoSMCs viability was conducted using WST-1 assay (Takara, Takara Bio Europe), which is based on the spectrophotometric measurements of formazan formed in viable cells. The amount of soluble product of mitochondrial dehydrogenase activity correlates with the number of living cells. HUVECs and AoSMCs were seeded at the density of 7500 and 5000 cells per well on 96-well plates, respectively. The cells were cultured for 24 h to obtain 70% confluency, followed by addition of compounds in medium at the appropriate concentrations varying from 0.001 to 3.0–10.0 μmol/mL. The cells were incubated with tested compounds or pure medium (control) for 24 h (37 °C, 5% CO_2_). On the next day, the cells were washed with 100 μL of culture medium (100 μL) and the reagent diluted in medium (1 + 9) was added. The plates were incubated at 37 °C in 5% CO_2_ for 90 min followed by the abosrbance read at 450 nm using a microplate reader (iMARK, Bio-Rad, Hercules, CA, US).

The experiments were conducted in multiplicates (*n* = 8 at least), and the results were presented as mean ± standard deviation (SD). Cellular viability was expressed as a percentage of the control samples which constituted 100% viability. IC_50_ values (the concentration of tested compound that inhibited cell growth by 50%) were calculated using concentration-response curves (GraphPad Prism, GraphPad Software, La Jolla, CA, US). The method was validated and the variability coefficient was counted (CV = 8.15%, *n* = 8).

Additionally, the effects towards HUVECs and AoSMCs was examined microscopically using inverted microscope with phase contrast (magnification 100×) (Opta-Tech, Warsaw, Poland, software OptaView 7).

### 3.5. AoSMCs Apoptosis

The preparation of samples for apoptosis assay included seeding the cells at the density of 50,000 per well on 24 well plate, 24-h incubation (37 °C, 5% CO_2_), followed by addition of medium (control) or medium with tested compounds at appropriate concentrations, and subsequent incubation for the next 24 h. Afterwards, the content of all wells was discarded, the cells were washed with DPBS, trypsynised, trypsin neutralizing solution was added, and the cells were collected to Eppendorf tubes. After centrifugation (220× *g*, 5 min) the cells were resuspended in cold cell staining buffer (BioLegend) and washed twice with this solution. Then the cells were suspended in 100 μL of binding buffer, and solutions of propidine iodide (PI) (10 μL) and FITC-Annexin (5 μL) were added. The samples were incubated for 20 min at room temperature in the dark, followed by the analysis conducted on cytometer (CytoFlex, blue laser, 480 nm, Beckman-Coulter, Indianapolis, IN, US). The results were analysed using Kaluza 2.1 (Beckman-Coulter) software.

The analysis of obtained results was made on the principle that annexin V(-) and PI (-) cells were considered as living cells, annexin V (+) and PI (-) as early-apoptotic cells, annexin V(+) and PI (+) as late-apoptotic cells, and annexin V (-) and PI (+) as necrotic cells. The results are presented as the percentage of the cells collected in gate B, and separated gate C. The method was validated and coefficients of variation were determined (CV = 0.62–4.58%, depending on the measured parameter, *n* = 4).

### 3.6. Migration Test

AoSMC cells were cultured on 24-well plates and incubated until reaching 70% confluence. After culturing for 24 h, the confluent cells were wounded by scratching with 100-μL pipette tip, and the wells were rinsed with 250 μL of fresh medium. Afterwards 250 μL of medium was replaced by the same volume of fresh medium (control) or medium including compounds at various concentrations. The plates were incubated up to 24 h at 37 °C (5% CO_2_) during which migration of cells was monitored using inverted microscope (Opta-Tech) with 4× objective. The images of cells migration were acquired at time intervals of 0, 2, 4, 8, and 24 h. The images were analyzed by microscope software (Opta-Tech Software), and the width of the scratch area was measured. Three independent experiments were performed in triplicates, and the results are presented as mean ± SD of the scratch width.

The coefficients of variation for the applied method were determined by two independent assays conducted in quadruplicate (CV = 9.36–14.72%, depending on the time point).

### 3.7. ICAM Expression on AoSMCs Surface

The preparation of cell samples was conducted according to the procedure applied in apoptosis test. The cells of each well were suspended in cold cell staining buffer (BioLegend), and solution of PE anti-human CD54 antibody was added. CD54 is a 85-110 kD type I transmembrane protein which is expressed on several types of cell, including leukocytes and endothelial cells. CD54 plays a role in cellular adhesion and is involved in inflammation and leukocyte extravasation. SMCs comprise the medial layer of the artery wall, and form a thick barrier. SMC cells are the source of proinflammatory cytokines and mediators, and participate in chronic inflammatory disorders in which both increased apoptosis and expression of ICAM-1 are observed. ICAM-1 expressed on aortal SMCs, serve as the adhesion molecule for leukocytes and facilitates smooth muscle cells migration [36].

The cells were incubated at room temperature for 20 min, and then analysed by flow cytometry (blue laser, 488 nm; Beckman-Coulter, CytoFlex). The results were analysed using the Beckman- Coulter Kaluza 2.1 software (Beckman-Coulter, Indianapolis, IN, US).

Controls using PE mouse IgG1 kappa isotype (BioLegend, UK) were also performed. The results are presented as the percentage of the total single cells gathered in gate A bound with CD54 antibody. The coefficients of variation for the method was counted (CV = 7.62%, *n* = 9).

### 3.8. Platelet Rich Plasma (PRP) for the Measurements of Platelets Aggregation and Adhesion

The studies using the biological material were approved by the Bioethics Committee of the Medical University of Lodz (RNN/350/18/KE). All subjects (volunteers) signed an informatory consent prior to participating in the study. The blood samples were collected from healthy donors (both sex, age range 18–50, not suffering from any chronic diseases, non-smoking, not taking anti-platelets drugs).

The blood was collected from the median cubital vein in the antecubital fossa into vacuum tubes containing 3.2% buffered sodium citrate (1:9) (Becton Dickinson, Franklin Lakes, New Jersej, US) for platelets analysis measurements. The blood samples were stored at room temperature up to 3 h before preparation of PRP.

Platelet-rich plasma (PRP) for platelet aggregation studies was obtained by blood centrifugation (150× *g*, 10 min, room temperature). To prevent the platelet activation, PRP was restored in a water bath at 37 °C for 30 min. The number of platelets in PRP was counted using turbidimetric method [52]. Obtained PRP was diluted twice with PBS (Biomed Lublin, Lublin, Poland), to obtain 250 000 platelets in 1 μL.

Platelets for adhesion analysis were isolated by centrifugation of the PRP (700× *g*, 15 min, RT) and suspending the platelets pellet in buffer A (pH 7.4; 145 mmol/L NaCl; 5 mmol/ L KCl; 10 mmol/L HEPES; 0.5 mmol/L Na_2_HPO_4_; 6 mmol/L glucose; 0.2% bovine serum albumin). The platelet count was performed by the method of Walkowiak et al. [52].

### 3.9. Platelet Aggregation Assay

Platelet aggregation in PRP was measured using turbidimetric method described elsewhere [53]. Briefly, aggregation curves were triggered by the addition of 10 μL ADP (10 μmol/L), recorded on Cecil CE2021 spectrophotometer with stirrer at 600 nm (Cecil, London, UK) and evaluated using computer software [53]. The following parameters were estimated: maximal aggregation (Amax), initial velocity (*v*_0_), the time needed to reach maximal aggregation (T_max_), the aggregation level after 5 min (A_5min_) from A_max_ (enables to estimate platelets disaggregation), and platelet shape change (PSC).

The coefficients of variation for this method was determined by four independent assays (CV = 8.90–10.39%, depending on the measured parameter).

### 3.10. Platelet Adhesion Assay

Spontaneous and ADP-activated platelet adhesion to human fibrinogen (2 mg/mL) coated 96-well microplates was investigated spectrophotometrically at 405 nm (microplate reader, iMARK, Bio-Rad) according to the previous protocol [53].

The content of each well was as follows, 45 µL of platelet suspension (final quantity 2.5 × 10^6^ platelets per well), 25 µL of buffer containing calcium and magnesium ions, 20 µL of 0.9% NaCl and 10 µL of tested compound. In the case of activated adhesion, 10 µL ADP (1 mmol/L) was added to stimulate platelets. Each sample had its own control (NaCl was added instead of the examined compound). After 1 h incubation at 37 °C, non-adhered platelets were carefully rinsed with 0.9% NaCl. Then, 150 µL of citrate buffer (pH = 5.4) containing reactions substrate (NPP) and Triton-X was added, and the plate was incubated for 1 h. Afterwards, 100 µL of sodium hydroxide was added to each well, and the absorbance was read at 405 nm. The density of yellow coloration was proportional to the activity of acid phosphatase in remained, adhered platelets.

The results (spontaneous and ADP-activated adhesion) are expressed as the percentage of the total number of platelets in every well, and were compared to respective control samples (spontaneous and ADP-induced). Three independent experiments were performed using three different blood donors, and were conducted in quadruplicates. For each experiment a calibration curve was prepared (R^2^ = 0.992–0.996). The coefficients of variation for this method was determined by two independent assays conducted in quadruplicate (CV = 14.82% for spontaneous adhesion, and CV = 13.93% for ADP-inducted adhesion).

### 3.11. Statistical Analysis

Statistical analysis was conducted using the commercially-available package (Statistica 12.0, StatSoft; GraphPad Prism 5). Data in tables and figures are presented as the mean ± standard deviation (SD) for variables with a normal distribution of values. The normal distribution of continuous variables was verified with the Shapiro-Wilk test, while homogeneity of variances was checked using the Levene test. The One-Way Analysis of Variance (ANOVA) was used to compare the means of independent groups (including control) in order to determine whether there was statistical evidence that the associated population means (tested compounds) were significantly different. The variables with non-normal distributions were compared using the Wilcoxon signed rank test. The results of all the tests were considered significant at *p*-values lower than 0.05.

## 4. Conclusions

Our findings suggest that metformin, a drug frequently used in the clinical management of T2DM patients, has beneficial effects on selected parameters of platelet and vascular haemostasis in vitro, including both endothelial and smooth muscle cells. Metformin does not influence the viability of ECs or AoSMC cells within the concentration range 0.006–3.0 μmol/mL. These results seem to be in agreement with those previously obtained using RTCA-DP [34]: metformin led to an insignificant increase in the cellular index (CI) in HUVEC cells, but this CI increase was only observed in the first six hours of analysis in the case of AoSMC cells. Metformin also does not induce apoptosis or necrosis up to 1.5 μmol/mL. Furthermore, the drug significantly decreases migration of AoSMCs, and expression of ICAM-1 molecules on their surface. Finally, metformin significantly reduces the initial velocity (*v*_0_) of ADP-induced platelet aggregation in vitro, thus presenting anti-aggregatory properties.

Our key findings show that in vitro tested sulfenamide and sulfonamide derivatives (**1**–**4**) are characterized by favourable properties with respect to platelet parameters, ICAM-1 expression and migration of AoSMC cells, similarly to the parent drug, metformin. Unlike compounds **1** and **2**, 4-nitro-benzenesulfonamide (**3**) and 2-nitrobenzenesulfonamide (**4**) derivatives of metformin do not exert unfavourable effects on both HUVEC and AoSMC viability up to 1,5–2.0 μmol/mL, and significantly reduce smooth muscle cell migration in wound healing assay. Furthermore, *p*- and *o*-nitro-benzenesulfonamides **3** and **4** significantly decrease in vitro platelet aggregation, and spontaneous and ADP-induced platelet adhesion to fibrinogen.

To conclude, both nitrobenzenemetformin sulfonamides (compounds **3** and **4**) exhibit promising activity regarding selected parameters of vascular and platelet haemostasis, which encourage further studies on the design and synthesis of new sulfonamide derivatives of metformin. This is supported by our previous results [34], which found these compounds to demonstrate anti-coagulation properties, manifested by prolonged platelet-dependent thrombus formation in semi-physiological conditions and decreased vWF release from ECs in vitro. The key results of this paper suggest that chemical modification of the biguanide scaffold into nitrobenzene sulfonamide may be associated with enhanced anti-coagulant properties, and encourage further studies using alternative, more advanced in vitro models (e.g., endothelial cells from diabetes donors) under hyperglycaemia conditions.

## Figures and Tables

**Table 1:** The effects of metformin derivatives on HUVEC and AoSMC cell growth. The results (IC_50_ values, µmol/mL) are presented as mean ± SD (*n* = 6–8).

Compound	HUVEC Cells [µmol/mL]	AoSMC Cells [µmol/mL]
**1**	3.781 ± 1.130	0.902 ± 0.015
**2**	1.044 ± 0.131	1.247 ± 0.062
**3**	2.407 ± 0.203	NE^#^
**4**	2.742 ± 0.119	NE^‡^

NE^#^: AoSMC cell viability was 79.33 ± 6.35% after 24-h incubation with 3.0 µmol/mL compound **3** (IC_50_ value was not estimated); NE^‡^: AoSMC cell viability was 62.19 ± 2.89% after 24-h incubation with 3.0 µmol/mL compound **4** (IC_50_ value was not estimated). Two-way ANOVA analysis revealed significant differences in cellular viability between HUVECs and AoSMCs for compounds **1**, **3** and **4**.

**Table 2:** Annexin V-FITC/PI double staining analysis of apoptosis in AoSMCs.

Compound [μmol/mL]	Single Cells in Gate B [%] ^1^		Living Cells [D - -] [%] ^2^	Necrotic Cells [D - +] [%] ^2^	Early Apoptotic [D + -] [%] ^2^	Late Apoptotic [D + +] [%] ^2^
**Control_1**	B	91.04 ± 0.81	91.20 ± 2.11	0.92 ± 0.25	4.78 ± 1.46	3.11 ± 0.87
**Met** **[0.3 μmol/mL]**	B	91.99 ± 0.03	91.81 ± 1.03	0.61 ± 0.11	4.02 ± 0.92	3.57 ± 0.23
**Met** **[1.5 μmol/mL]**	B	91.82 ± 0.43	92.13 ± 0.57	0.55 ± 0.09	3.77 ± 0.10	3.55 ± 0.42
**Comp. 1** **[0.1 μmol/mL]**	B	92.70 ± 0.41	91.36 ± 2.59	0.60 ± 0.05	4.71 ± 1.95	3.33 ± 0.68
**Comp. 1** **[0.9 μmol/mL]**	B	**61.36 ± 1.15**	**12.33 ± 2.03**	0.55 ± 0.15	**71.36 ± 2.55**	**15.76 ± 0.61**
**Control_2**	B	92.45 ± 0.58	92.34 ± 2.02	0.90 ± 0.41	4.05 ± 0.91	2.71 ± 0.82
**Comp. 2** **[0.3 μmol/mL]**	B	92.85 ± 0.67	89.26 ± 3.13	0.70 ± 0.20	4.82 ± 1.48	4.70 ± 1.63
**Comp. 2** **[1.0 μmol/mL]**	B	92.19 ± 0.60	**83.84 ± 0.47**	0.90 ± 0.23	**7.96 ± 0.43**	**7.30 ± 0.63**
**Comp. 3** **[0.3 μmol/mL]**	B	94.32 ± 0.43	90.83 ± 1.78	0.83 ± 0.15	4.98 ± 1.40	3.37 ± 0.63
**Comp. 3** **[1.5 μmol/mL]**	B	94.20 ± 0.47	**89.23 ± 2.55**	0.99 ± 0.22	5.69 ± 1.92	4.08 ± 0.73
**Comp. 4** **[0.3 μmol/mL]**	B	93.79 ± 0.52	**88.84 ± 0.21**	1.05 ± 0.22	**5.11 ± 0.41**	5.01 ± 0.13
**Comp. 4** **[1.5 μmol/mL]**	B	93.08 ± 0.45	**82.61 ± 0.97**	1.58 ± 0.26	**8.29 ± 0.35**	**7.52 ± 0.56**

Aortal smooth muscle cells were treated with various concentrations of biguanides for 24 h followed by staining with Annexin V FITC and propidine iodide (PI). The concentration for apoptosis assay were chosen on the basis of results collected in viability test. ^1^**–**Single cells gathered within the gate B reflecting the % of the absolute number of acquired events; ^2^–the single cells gathered in gate B were divided depending on staining with Annexin V and PI: (D- -) - living cells, (D - +) – necrotic cells; (D + -) – early-apoptotic cells; (D + +) – late-apoptotic cells. The results are presented as mean ± standard deviation (SD), n = 3. The values given in bold represent statistically significant (*p* < 0.05) changes versus respective controls (metformin, and compound **1**–control_1; compounds **2**–**4**–control_2). Two-way Anova analysis showed significant differences in AoSMC cell viability and apoptosis between compound **1** (the most profound apoptosis induction) and all other compounds (**2**–**4**).

## Supplementary Materials

Supplementary materials can be found online. Figure S1: The effects of sulfenamide and sulfonamide derivatives of metformin on selected parameters of plasma and vascular haemostasis, Synthesis protocol for compounds **1**–**4**, Spectroscopic characterization of compounds **1**–**4**, Figure S2: The effects of compounds **1**–**4** on the viability of HUVEC cells, Figure S3: The effects of compounds **1**–**4** on the viability of AoSMC cells, Figure S4: Inhibition of cell migration in the presence of biguanides, Table S1: The effects of metformin and its derivatives on AoSMC cells migration. Table S2: The effects of biguanides on the expression of CD54 (ICAM-1) on the surface of smooth muscle cells. Table S3: In vitro effects of biguanide derivatives on kinetic parameters of ADP-induced platelet aggregation, Figure S5: The characteristic of HUVEC cells: ICAM-1 expression on the cell surface and staining with Annexin V and propidine iodide.
