# Supplementary material for: Pleiotropic Activity of Metformin and Its Sulfonamide Derivatives on Vascular and Platelet Haemostasis
Source: Molecules. 2019 Dec 28;25(1):125. doi: 10.3390/molecules25010125 (PMC6982810; doi:10.3390/molecules25010125)
Supplement: Supplementary file 1 [file molecules-25-00125-s001.pdf]

**Pleiotropic activity of metformin and its sulfonamide derivatives on vascular and platelet haemostasis"**

**Magdalena Markowicz-Piasecka, Kristiina M. Huttunen, Adrianna Sadkowska, Joanna Sikora**

***Figure S1.*** *The effects of sulfenamide and sulfonamide derivatives of metformin on selected parameters of plasma and vascular haemostasis*.*

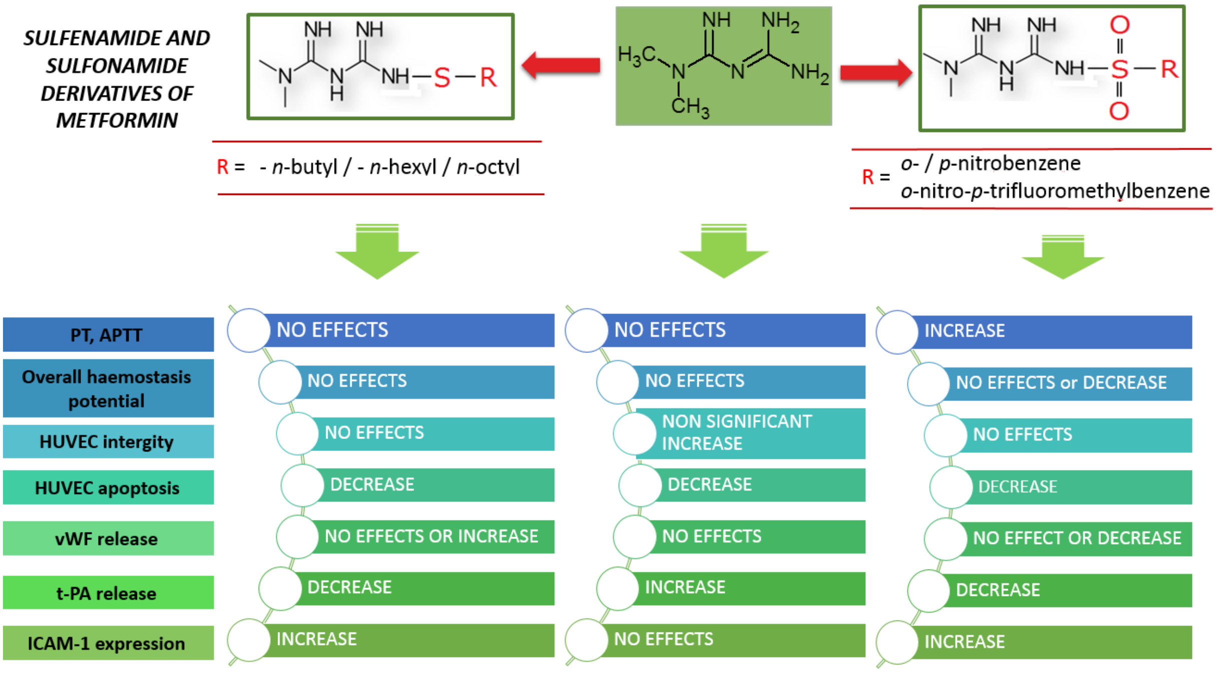


* Based on the results of our previous papers; Markowicz-Piasecka et al., 2017, Eur J Pharm; Markowicz-Piasecka et al., 2018, Chem-Biol Int; Markowicz-Piasecka et al., 2019, Sci Rep.

## Synthesis protocol for compounds 1 – 4

Metformin (N,N-dimethyl imidodicarbonimidic diamide hydrochloride) (1.0 eq.) in 1 M NaOH (1.5 eq.) was stirred at room temperature for 30 min. Water was evaporated in vacuo and the residue was dissolved in MeOH. The solvent was evaporated and the residue was redissolved in cold anhydrous MeOH. NaCl was filtered out of the solution and the filtrate was evaporated to yield basic metformin as a white solid (99%).

<u>Compound 1</u>

Basic metformin (2.0 eq.), $AgNO_3$ (1.0 eq.) and disulfide (1.0 eq.) were dissolved in anhydrous MeOH in a sealed pressurerated glass tube and irradiated at 80 ºC in a microwave reactor for 30 min. The reaction mixture was filtered and the filtrate was treated by AcOH (2.2 eq.) or 1 M HCl (1.1 eq.). The solvent was removed under reduced pressure and the residue was purified by preparative HPLC on Kromasil 100 C8 column eluting with 0.1% AcOH solution and acetonitrile (15:85, v/v).

Compounds 2 – 4
Basic metformin (2.0 eq.), and commercial sulfonyl chlorides (1.0 eq.) were dissolved in anhydrous $CH_2Cl_2$ and stirred under argon for 3 h. The solvent was evaporated under reduced pressure and the residue was purified by flash column chromatography eluting with 0-30% MeOH in $CH_2Cl_2$ to obtain the compounds **1-3** (19-72%).

**Spectroscopic characterization of compounds 1 – 4**

$N^1$,$N^1$-dimethyl-$N^4$-(butylthio)-bisguanidine (**1**)
$^1$H NMR ($CD_3OD$): δ ppm 3.04 (s, 6H), 2.75 (t, 3JHH = 7.4 Hz, 2H), 1.68–1.60 (m, 2H), 1.51–1.43 (m, 2H), 0.95 (t, 3JHH = 7.4 Hz, 3H); $^{13}$C NMR ($CD_3OD$): δ ppm 161.45, 160.62, 40.02, 38.06 (2C), 30.94, 22.72, 14.02.
MS (ESI+) for $C_8H_{20}N_5S$ (M)+: Calcd 218.33, Found 218.13.

$N^1$,$N^1$-Dimethyl-$N^4$-(2-nitro-4-(trifluoromethyl)benzenesulfonamide)-bisguanidine (**2**)
Prepared from 2-nitro-4-(trifluoromethyl)benzenesulfonyl chloride.
$^1$H NMR ((CD$_3$)2SO): δ ppm 7.45 (s, 1H), 8.28 (d, $^3J$HH=8.2 Hz, 1H), 8.16 (d, $^3J$HH=8.2 Hz, 1H), 8.02-7.88 (bs, 2H), 7.24-6.62 (bs, 2H), 2.92 (s, 6H); $^{13}$C NMR ($CDCl_3$): δ ppm 158.93, 158.45, 147.49, 139.45, 132.53, 130.37, 128.80, 123.64, 121.49, 36.69 (2 C).
MS (ESI+) for $C_{11}H_{14}F_3N_6O_4S$ (M+H)+: Calcd 383.33, Found 383.03. Anal. Calcd for ($C_{11}H_{13}F_3N_6O_4S$): C, 34.56; H, 3.43; N, 21.98; S, 8.39; Found: C, 34.76; H, 3.09; N, 21.57; S, 8.20.

$N^1$,$N^1$-Dimethyl-$N^4$-(4-nitrobenzenesulfonamide)-bisguanidine (**3**)
Prepared from 4-nitrobenzenesulfonyl chloride. $^1$H NMR ($(CD_3)_2SO$): δ ppm 8.33 (d, $^3J$HH=8.8 Hz, 2H), 8.02 (d, $^3J$HH=8.8 Hz, 2H), 8.08-7.79 (bs, 2H), 7.20-6.94 (bs, 1H), 6.90-6.62 (bs, 1H), 2.91 (s, 6H); $^{13}$C NMR ($(CD_3)_2SO$): δ ppm 159.52, 158.32, 149.80, 148.88, 127.30 (2 C), 124.24 (2 C), 36.61 (2 C).
MS (ESI+) for $C_{10}H_{15}N_6O_4S$ (M+H)+: Calcd 314.33, Found 315.10. Anal. Calcd for ($C_{10}H_{14}N_6O_4S$): C, 38.21; H, 4.49; N, 26.74; S, 10.20; Found: C, 38.32; H, 4.19; N, 26.59; S, 9.96.

$N^1$,$N^1$-Dimethyl-$N^4$-(2-nitrobenzenesulfonamide)-bisguanidine (**4**)
Prepared from 2-nitrobenzenesulfonyl chloride. $^1$H NMR ($(CD_3)_2SO$): δ ppm 8.09-8.04 (m, 1H), 8.29-7.88 (bs, 2H), 7.87-7.83 (m, 1H), 7.78-7.74 (m, 2H), 7.30-6.92 (bs, 1H), 6.91-6.52 (bs, 1H), 2.93 (s, 6H); $^{13}$C NMR ($(CD_3)_2SO$): δ ppm 159.07, 158.38, 147.38, 135.71, 132.90, 131.87, 128.90, 123.85, 36.62 (2 C).
MS (ESI+) for $C_{10}H_{15}N_6O_4S$ (M+H)+: Calcd 314.33, Found 315.10. Anal. Calcd for ($C_{10}H_{14}N_6O_4S$): C, 38.21; H, 4.49; N, 26.74; S, 10.20; Found: C, 38.56; H, 4.09; N, 26.37; S, 10.35.

***Figure S2.*** *The effects of compounds* ***1 - 4*** *on the viability of HUVEC cells. The cells were incubated with compounds at various concentrations (0.006 – 10.0 µmol/mL) for 24 hours, and the viability of the cells was measured spectrophotometrically using the WST-1 test. The results are presented as mean ± SD, n = 6 - 8. One-way Anova analysis revealed significant differences in HUVEC viability at various concentrations (* p < 0.05; ** p < 0.01; *** p < 0.001).*

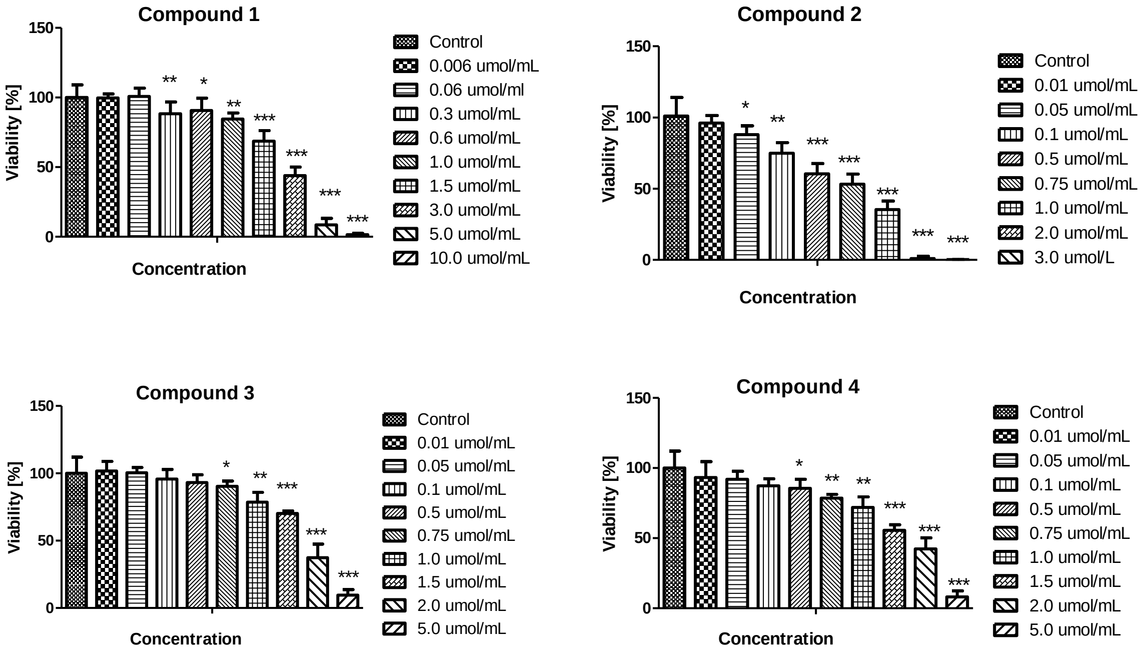

***Figure S3.*** *The effects of compounds 1 - 4 on the viability of AoSMC cells. The cells were incubated with compounds at various concentrations (0.006 – 3.0 μmol/mL) for 24 hours, and the viability of the cells was measured spectrophotometrically using the WST-1 test. The results are presented as mean ± SD, n = 8. One-way Anova analysis revealed significant differences in AoSMCs viability at various concentrations (* p < 0.05; ** p < 0.01; *** p < 0.001).*

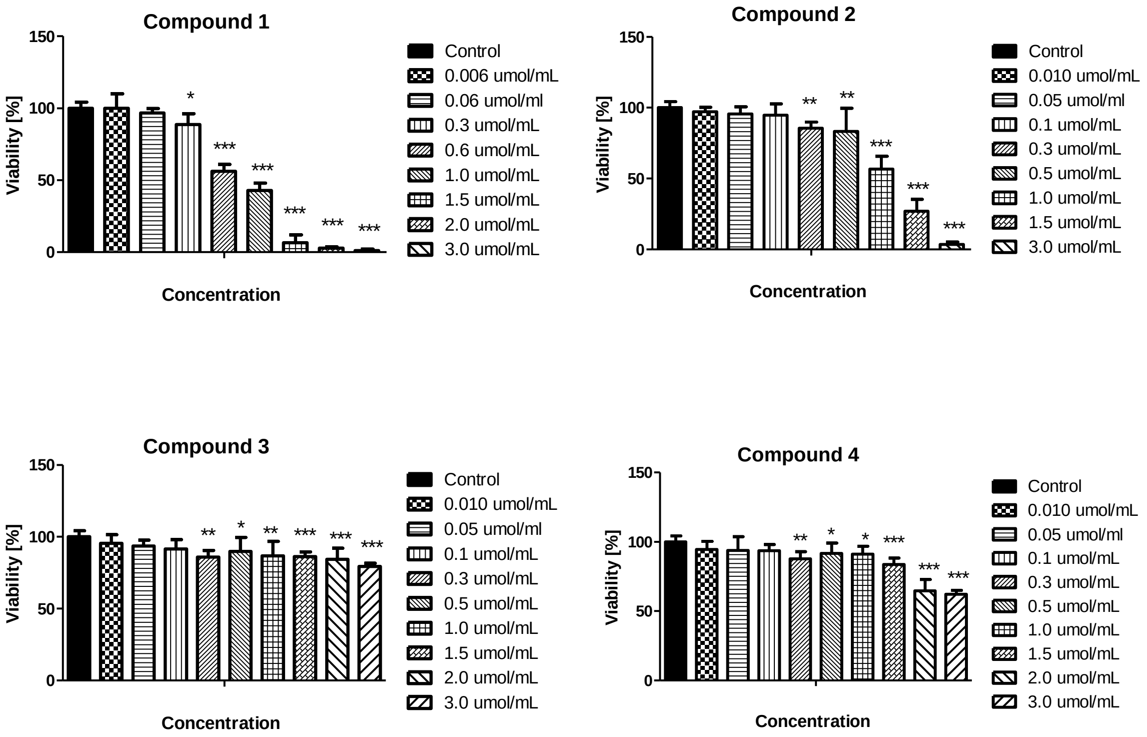


***Figure S4.*** *Inhibition of cell migration in the presence of biguanides. AoSMC cells migration was evaluated using wound healing assay. Representative cell images are shown for control samples and*

*biguanides at the concentration of 1.5 (metformin) or 1.0 μmol/mL (compounds 1-4). Cells were photographed using an inverted microscope at the indicated times (t = 0, 8, 24 h); 100× magnification.*

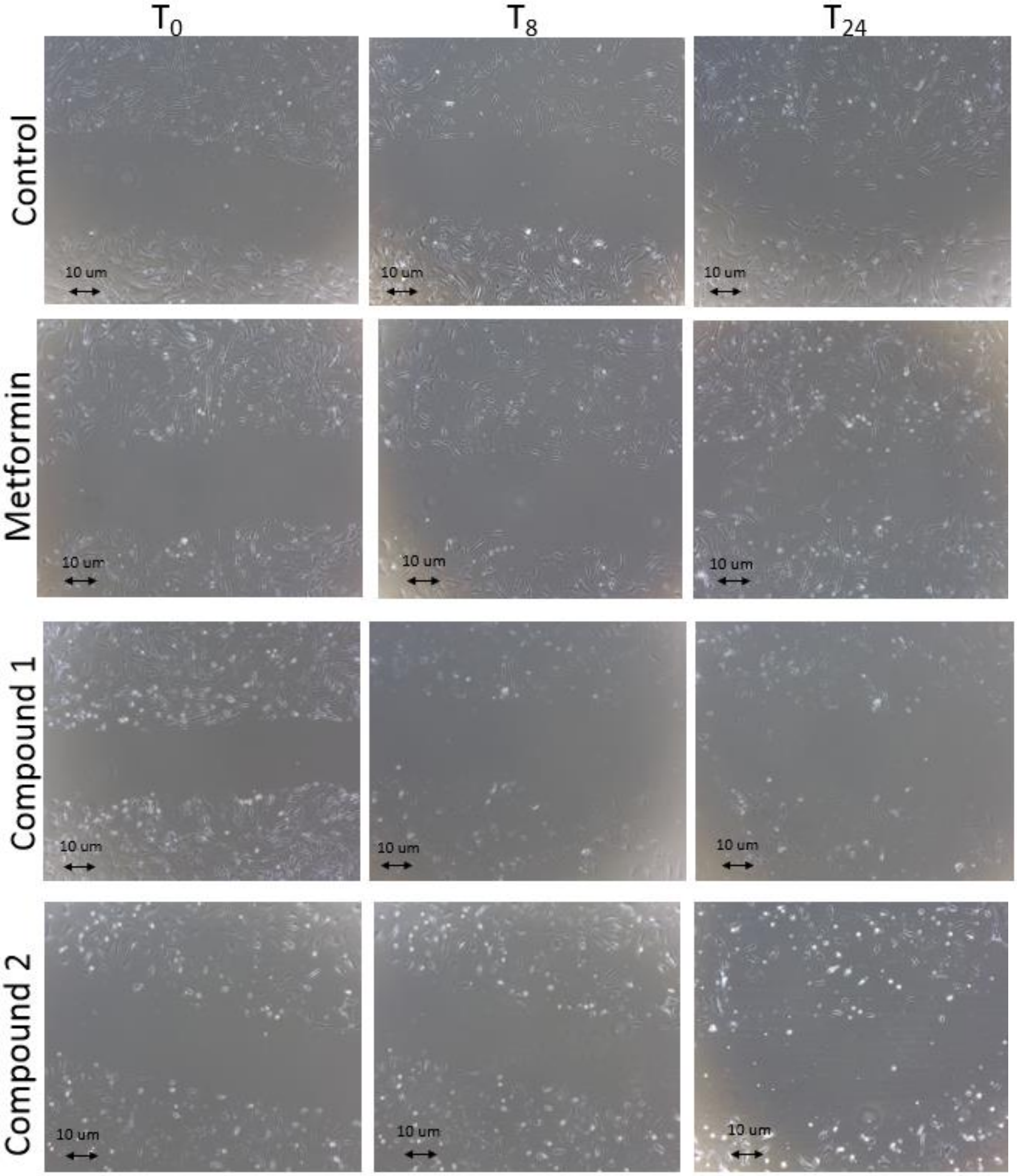

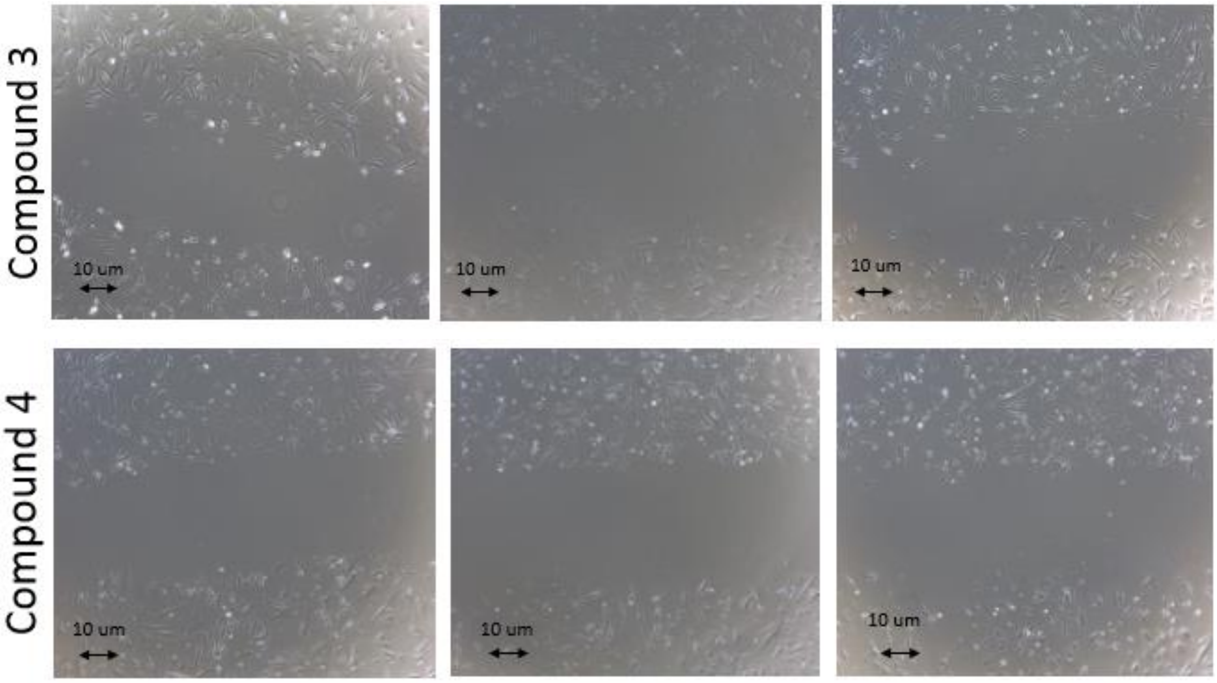


***Table S1.*** *The effects of metformin and its derivatives on AoSMC cells migration.*

| Compound | Conc. [µmol/mL] | $T_0$ [µm] | $T_2$ [µm] | $T_4$ [µm] | $T_8$ [µm] | $T_{24}$ [µm] |
|---|---|---|---|---|---|---|
| **Control** | - | 62.49 ± 8.93 | 55.91 ± 10.29 | 52.56 ± 10.54 | 45.86 ± 7.76 | 20.96 ± 2.82 |
| **Metformin** | 0.06 | 60.14 ± 8.94 | 61.31 ± 6.05 | 59.96 ± 7.18 | **55.63 ± 8.75** | **33.57 ± 5.50** |
| | 0.3 | 64.69 ± 6.63 | 61.95 ± 6.58 | **63.39 ± 5.54** | **58.14 ± 5.38** | **36.81 ± 6.24** |
| | 1.0 | 63.33 ± 5.86 | 63.12 ± 6.22 | **62.85 ± 5.26** | **60.15 ± 2.85** | **37.15 ± 7.76** |
| | 1.5 | 68.56 ± 12.96 | **66.83 ± 12.41** | **66.14 ± 11.42** | **63.32 ± 13.67** | **36.07 ± 6.12** |
| **Comp. 1** | 0.06 | 65.99 ± 11.09 | **66.29 ± 12.59** | 60.34 ± 11.03 | 54.07 ± 13.48 | **34.94 ± 10.83** |
| | 0.3 | 71.45 ± 13.22 | **70.68 ± 10.14** | **72.65 ± 11.12** | **66.46 ± 13.48** | **50.95 ± 10.43** |
| | 1.0 | 71.15 ± 9.48 | **68.46 ± 11.59** | **71.50 ± 10.99** | **72.46 ± 12.06** | **67.98 ± 8.06** |
| **Comp. 2** | 0.06 | 54.69 ± 5.17 | **50.14 ± 3.60** | 50.73 ± 7.94 | 46.81 ± 6.19 | **33.21 ± 5.14** |
| | 0.3 | 60.63 ± 6.85 | 62.70 ± 7.42 | 59.51 ± 9.04 | 57.02 ± 6.86 | **30.56 ± 9.00** |
| | 1.0 | 63.23 ± 5.96 | **67.82 ± 8.76** | **66.36 ± 5.99** | **65.87 ± 8.77** | **63.63 ± 8.04** |
| **Comp. 3** | 0.06 | 65.29 ± 5.44 | **64.71 ± 6.16** | **60.62 ± 7.93** | **57.99 ± 6.41** | **32.26 ± 8.49** |
| | 0.3 | 71.49 ± 9.28 | **77.54 ± 9.17** | **74.28 ± 6.92** | **72.64 ± 5.70** | **46.38 ± 10.36** |
| | 1.0 | 72.65 ± 7.37 | **77.57 ± 8.60** | **78.60 ± 8.32** | **72.93 ± 10.98** | **61.96 ± 8.92** |
| **Comp. 4** | 0.06 | 65.77 ± 12.17 | 63.99 ± 6.76 | 59.49 ± 6.15 | **57.92 ± 7.94** | **38.33 ± 11.14** |
| | 0.3 | 69.91 ± 10.50 | **68.31 ± 5.42** | **69.83 ± 8.52** | **65.24 ± 7.76** | **47.14 ± 7.18** |
| | 1.0 | 71.82 ± 9.55 | **71.36 ± 3.25** | **77.32 ± 5.74** | **73.30 ± 7.99** | **64.02 ± 5.67** |

The results are presented as mean ± standard deviation (SD) of the wound width, n = 10-12 at various time points ($T_0$, 2 hours ($T_2$), 4 hours ($T_4$), 8 hours ($T_8$) and 24 hours ($T_{24}$)). The values given in bold represent statistically significant ($p < 0.05$) changes versus control.

***Table S2.*** *The effects of biguanides on the expression of CD54 (ICAM-1) on the surface of smooth muscle cells.*

| **Compound** | **Conc. [μmol/mL]** | **ICAM-1 expression [%]** |
|---|---|---|
| **Control** | - | 5.44 ± 1.21 |
| **Metformin** | 0.3 | 5.04 ± 0.59 |
| | 1.5 | **4.21 ± 0.90** |
| **Comp. 1** | 0.1 | **4.98 ± 0.88** |
| | 0.3 | 5.19 ± 0.39 |
| **Comp. 2** | 0.3 | 3.06 ± 0.51 |
| | 1.0 | 4.84 ± 2.19 |
| **Comp. 3** | 0.3 | 3.17 ± 1.08 |
| | 1.5 | 4.86 ± 2.18 |
| **Comp. 4** | 0.3 | 4.44 ± 1.32 |
| | 1.5 | **11.34 ± 2.46** |

Smooth muscle cells were treated with various concentrations of biguanides for 24 hours followed by staining with PE-CD54 antibody. The concentration for the studies were chosen on the basis of results collected in viability assay. The results are presented as mean ± standard deviation (SD), n = 3-6. The values given in bold represent statistically significant ($p < 0.05$) changes versus respective controls.

***Table S3.*** *In vitro effect of biguanide derivatives on kinetic parameters of ADP-induced platelet aggregation (mean ± SD; n = 4 - 5).*

| | | AGGREGATION PARAMETERS | | | | |
|---|---|---|---|---|---|---|
| **Compound** | **Concentration [μmol/mL]** | **Amax [%T]** | **Tmax [s]** | **PSC [%T]** | **$V_0$ [%T/min]** | **$A_{5min}$ [%T]** |
| **Control** | - | 57.30 ± 8.07 | 236.82 ± 38.30 | 2.04 ± 1.12 | 56.05 ± 8.78 | 58.38 ± 8.92 |
| **Metformin** | 0.06 | 51.58 ± 8.06 | 224.00 ± 20.61 | 1.79 ± 1.47 | **46.90 ± 7.14*** | 51.43 ± 7.91 |
| | 0.3 | 47.90 ± 8.89 | 233.25 ± 45.24 | 2.64 ± 2.02 | **44.70 ± 4.21**** | 48.55 ± 10.21 |
| | 1.0 | 47.91 ± 10.15 | 230.14 ± 41.77 | 2.59 ± 1.09 | **45.27 ± 2.31**** | 50.76 ± 9.83 |
| | 1.5 | 47.45 ± 9.59 | 228.25 ± 49.43 | 2.64 ± 1.55 | **44.83 ± 5.33*** | 51.48 ± 13.36 |
| **Compound 1** | 0.06 | 53.50 ± 7.80 | 227.75 ± 21.56 | 2.08 ± 1.17 | 44.50 ± 6.46 | 53.50 ± 7.84 |
| | 0.3 | 43.17 ± 13.62 | 218.25 ± 48.63 | 3.43 ± 3.79 | 44.05 ± 14.99 | 46.80 ± 13.62 |
| | 1.0 | **31.33 ± 8.41*** | 233.00 ± 81.10 | 2.57 ± 1.83 | **26.68 ± 7.19**** | **31.08 ± 8.87*** |
| **Compound 2** | 0.06 | 59.33 ± 5.53 | 215.25 ± 30.24 | 0.69 ± 0.25 | 47.24 ± 5.84 | 59.28 ± 5.52 |
| | 0.3 | 50.69 ± 7.70 | **192.75 ±27.22**** | 1.64 ± 0.41 | 46.18 ± 9.66 | 50.45 ± 7.71 |
| | 1.0 | 31.25 ± 16.96 | **96.28 ± 41.36**** | 1.63 ± 0.99 | 34.30 ± 15.68 | **28.74 ± 15.54*** |
| **Compound 3** | 0.06 | 55.63 ± 9.43 | 208.75 ± 12.39 | 0.82 ± 0.40 | **46.35 ± 3.80*** | 55.60 ± 9.43 |
| | 0.3 | 58.48 ± 2.43 | 219.25 ± 17.73 | 1.02 ± 0.76 | 48.88 ± 2.97 | 58.00 ± 5.74 |
| | 1.0 | 50.43 ± 3.07 | 177.50 ± 17.21 | 2.04 ± 0.58 | **45.50 ± 0.93**** | **47.50 ± 5.75*** |
| **Compound 4** | 0.06 | 57.40 ± 3.81 | 225.75 ± 19.52 | 1.37 ± 0.28 | 45.23 ± 6.42 | 57.35 ± 3.85 |
| | 0.3 | 54.30 ± 4.29 | 211.50 ± 19.23 | 2.25 ± 1.29 | 46.88 ± 6.84 | 54.33 ± 4.25 |
| | 1.0 | **44.13 ± 4.73*** | 196.50 ± 29.69 | 3.21 ± 0.97 | **42.08±1.44***** | **44.30 ± 4.80**** |

Amax (%T) maximal aggregation, Tmax (s) time needed to reach Amax, $v_0$ (%T/min) initial velocity, and PSC (%T) maximal platelet shape change, $A_{5min}$ the aggregation level after 5 min. * $p < 0.05$; ** $p < 0.01$; *** $p < 0.001$.

***Figure S5.*** *The characteristic of HUVEC cells: ICAM-1 expression on the cell surface and staining with Annexin V and propidine iodide.*

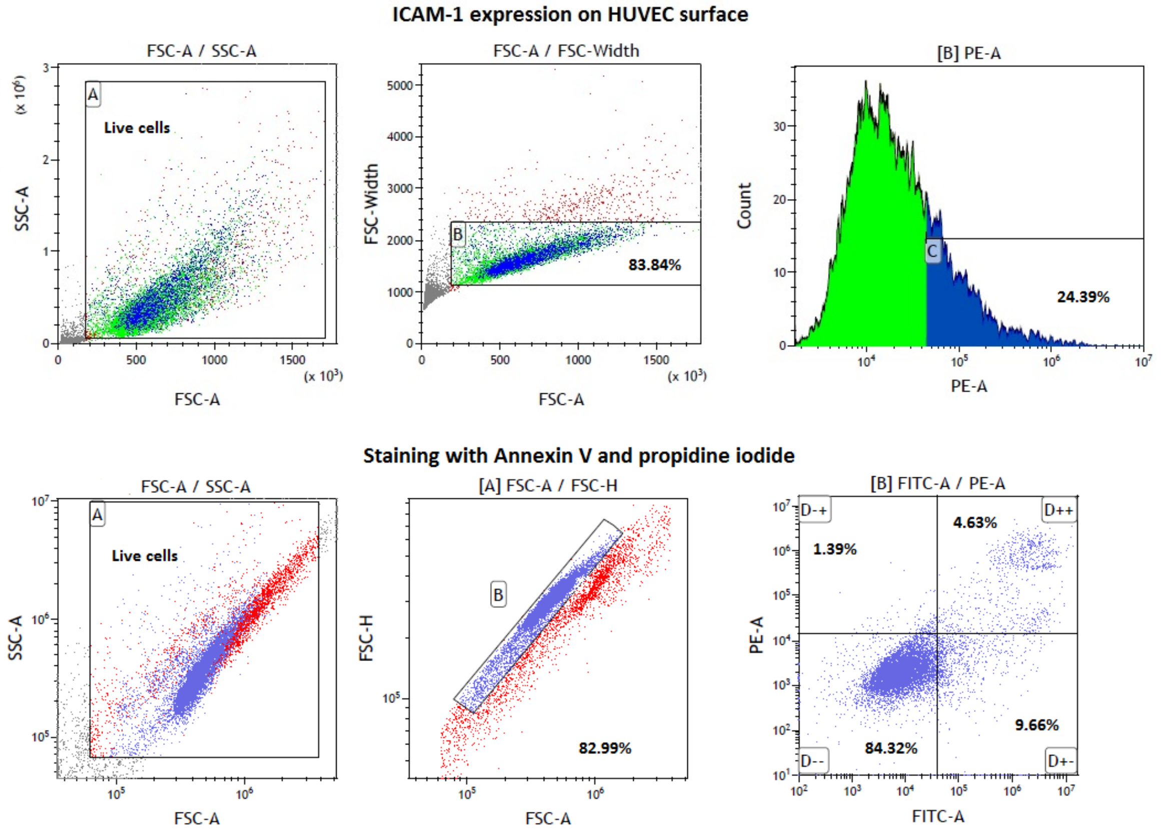


**ICAM-1 expression on HUVEC surface:** Representative histograms of unstimulated HUVEC cells (control). Cytogram on the left side: Forward and side scatter plot of HUVEC cells. The living cells were marked with gate A. Cytograms in the centre: a forward scatter width (FSC-W) vs. forward scatter area (FSC-A) of the cells gathered in gate A. Singe cells were marked with gate B. Cytograms on the right side: percentage of single cells expressing ICAM-1.

**Viability test using double staining with Annexin V and priopidine Iodide:** Representative histograms of unstimulated HUVEC cells (control). Cytogram on the left side: Forward and side scatter plot of HUVEC cells. The living cells were marked with gate A. Cytograms in the centre: a forward scatter width (FSC-W) vs. forward scatter area (FSC-A) of the cells gathered in gate A. Singe cells were marked with gate B (ca. 82.99%). Cytograms on the right side: Annexin V FITC-A (x-axis) vs propidium iodide (y-axis) plots from the gated cells (B) show the populations corresponding to living cells (Annexin V(-) and PI (-)) (D- -) – 84.32%; early apoptotic cells (Annexin V (+) and PI (-)) (D+-) – 9.66%, late-apoptotic cells (Annexin V(+) and PI (+)) (D++) – 4.63%, and necrotic cells (Annexin V (-) and PI (+)) (D-+) – 1.39%.
